# Policy integration, policy coherence, and whole-of-government approaches: a qualitative systematic review of advice for policymakers

**DOI:** 10.12688/openreseurope.19864.2

**Published:** 2026-07-09

**Authors:** Paul Cairney

**Affiliations:** 1 Division of History, Heritage and Politics, University of Stirling, Stirling, Scotland, FK94LA, UK

**Keywords:** policy, policymaking, governance, wicked problems, policy integration, policymaking integration, policy coherence, whole-of-government, joined-up-government

## Abstract

**Background:**

The pursuit of policy integration and policy coherence represents the ultimate gap between aspiration and reality. Policy processes are too fragmented to produce coherent policies to address complex problems. Whole-of-government and joined-up-government are popular buzzwords that struggle for meaning and traction. How can policymakers and researchers address this problem? I searched multiple academic literatures for advice on how to improve policy integration and coherence.

**Methods:**

A qualitative systematic review of 705 academic journal articles identified in two systematic reviews on ‘whole-of-government and joined-up government’ (WG/JUG, 340) and ‘policy integration’ (PI, 413), and snowballed texts in academic and grey literatures (65) (net total 770). I searched each text for advice on how to foster policy process integration to boost policy coherence. I used an immersive inductive approach, and policy theory insights, to identify common themes.

**Results:**

Most accounts describe aspirations for integration and coherence. Few describe substantive progress. The literature describes *requirements* for integration and coherence and inevitable or routine policymaking *barriers.* This comparison of barriers and facilitators helps to inform a realistic approach, to combine managed expectations and practical advice.

**Conclusions:**

Five themes of practical advice connect aspiration to limited progress. Explain what policy integration means, since a rhetorical commitment means nothing. Identify your rationale, model, and theory of change. Engage with trade-offs between top-down and bottom-up conceptions of policy coherence. Explain why the pursuit of integration has advantages over a reasonable alternative, such as specialisation. Learn about facilitators from studies of success and barriers from studies of failure. These lessons help to clarify your aims, connect them to routine government business, and ensure capacity to deliver. This advice may seem obvious in theory, but lacks traction in practice when governments lack the willingness and ability to follow it.

## Plain language summary

Terms like ‘whole-of-government’, policymaking ‘integration’, and ‘policy coherence’ describe what governments *require.* Complex problems are not amenable to simple solutions. Collaboration is necessary across government when problems transcend sectoral and jurisdictional boundaries. Collaboration outside of government is essential to democracy and effectiveness. In the absence of holism, policy would be made by many actors for different purposes, exacerbating incoherence.

However, there is a large gap between requirement and reality. Policymaking is too fragmented to produce fully integrated policymaking and coherent policies. Whole-of-government approaches are vague and rarely translated into concrete action.

There is limited agreement on how to respond. The pursuit of holism and coherence is ambiguous, approaches are difficult to track, and perspectives are difficult to reconcile. Most studies restate the importance of integrated action without defining it well, describing how to do it, addressing trade-offs, or evaluating progress. Research is not well integrated, and the result is not coherent. A search for advice is akin to seeking needles in haystacks spread across many fields.

In that context, my aim is to synthesise research insights to identify the models and practices associated with successful integration and coherent policy. I present a cautionary-tale-based approach built around five themes.

1.    Explain what policymaking integration means, since a rhetorical commitment alone means nothing.

2.    Identify your rationale, preferred model, and theory of change.

3.    Engage with trade-offs between top-down and bottom-up conceptions of policy coherence.

4.    Explain why the pursuit of integration has advantages over a reasonable alternative, such as to benefit from specialisation.

5.    Learn about facilitators from studies of success and barriers from studies of limited progress.

This advice may seem obvious, so why don’t governments do these things already? The review identifies their limited *willingness* and *ability* to produce clear and well-resourced integration.

## Introduction

The umbrella term *policy integration* shelters a collection of hopes for better coordinated policy processes and more coherence policies. We need better forms of coordination because complex policy problems transcend traditional policy sectors and jurisdictions and there is no single centre of authority. We need to pursue a coherent policy mix to anticipate the ability of many jurisdictions to produce their own instruments, which contribute to a messy incoherent mix of policies. Yet, political science research shows that there is a major and persistent gap between requirements versus policymaking reality. It uses a wide range of policy integration concepts to describe the often vague and rhetorical commitment to coordination and coherence and explain major gaps between stated commitments and real-world outcomes. While this academic case is convincing, it is not entirely helpful: policymakers do not have the luxury of enjoying a well-explained lack of progress, and still face the need to produce better coordinated processes and more coherent policies. It is essential to take this imperative seriously and establish how a wealth of policy integration research can help to guide real world action.

To that end, I present a qualitative systematic review of advice for *policy integration in the pursuit of policy coherence.* I draw on studies sheltering under that umbrella description, including whole-of-government and joined-up government approaches ( Aoki *et al.*, 2024a), policy integration, holistic governance, intersectoral action, policy coordination, and mainstreaming ( Trein *et al.*, 2023). I synthesise their insights to strengthen the connection between research and advocacy in policy analysis: academic research should be relevant to real-world policymaking and policymaking should be informed by academic research.

This review is also part three of a series that seeks advice on collaborative policymaking more generally ( Cairney & Toomey, 2024; Cairney & Toomey, 2025). It seeks usable evidence to learn what makes policymaking more effective, while reflecting on contestation on how to learn and to define what is effective. Policy learning is part of the politics of policymaking, not a technical exercise to identify optimality ( Cairney *et al.*, 2024; Dunlop & Radaelli, 2013; Rietig & Perkins, 2018). For example, in this review my aims *seem* like valence issues: who would *not* want holism, integrated policymaking, and coherent policy? However, they are misleadingly attractive, too ambiguous to be useful, and potentially damaging to progress. When prompted to be more precise, there are good reasons to expect contested ways to integrate policymaking, including:

1. A top-down approach, such as to concentrate power in a few central government actors who would oblige others to integrate.
2. A bottom-up approach, such as to distribute power and rely on voluntary action to collaborate. Decentralise responsibilities, allowing each authority to innovate and learn, and accept that they could go their own way.

In other words, one author’s fragmentation and incoherence is another’s legitimate division of powers and freedom to tailor policy to context. Indeed, the starting point for each of the three reviews is the frequent rejection of top-down central control as not possible or desirable ( Hooghe & Marks, 2003). Impossibility relates to complex policy problems, which ‘do not respect traditional government boundaries, and require collaborative responses across government and between governmental and non-governmental actors’ ( Cairney, 2024: 4). Undesirability relates to a normative alternative to distribute policymaking responsibilities to reflect: legitimate demands for subnational political autonomy, the value of specialist and semi-autonomous policymaking organisations, and the drive to incorporate local citizens and stakeholders in policymaking ( Cairney *et al.*, 2019, 2022a). When policymaking responsibilities are distributed across sectors and political systems, *some* may seek central control to prioritise the coherence of a policy mix. Others are searching for advice on more voluntary collaborative ways to improve policymaking and coherence while protecting their autonomy. Resolving this ambiguity involves exercising power rather than consulting a dictionary.

Such contestation informs this three-part series’ search for advice, to connect aspirations to theory-informed research. Aspirational stories involve different elements, such as rationalist imperatives towards centralised coherence or alternative democratic imperatives that favour decentralisation and voluntary collaboration. Therefore, policy theory-informed studies inform different aspects of the gap between our requirements of policymaking versus reality, such as the inability of: one governing centre to assert its control in a complex system, or a collaborative initiative to succeed on its own terms. Different stories create different demands for, and interpretations of, advice on how to make policymaking more integrated and policy more coherent.

This is the most ambitious of the reviews (of 770 texts). I make it manageable as follows. First, drawing on established reviews without performing new database searches. Key scholars have performed the initial stages, to identify large literatures (705 texts) as part of their systematic reviews: Aoki *et al.* (2024a, 2024b) on ‘whole-of-government and joined-up government’ (WG/JUG) and Trein *et al.* (2023) and Trein (2022) on ‘policy integration’ (PI) (I now use PI and WG interchangeably unless stated otherwise). Conducting another *search* would be inefficient but additional *analysis* is essential. Second, I seek insights from grey literatures, via snowballing not database search, based on previous dispiriting experiences of searching in grey literature databases when reports use aspirational policymaking terms loosely ( Cairney & Toomey, 2024, 2025).

I use the same approach to analyse the data, report, and reflect on the results. My search is for ‘pragmatic advice that offers hope’ but acknowledges policymaking constraints ( Cairney & Toomey, 2024: 4). I cast a wide net to catch any relevant advice, including ‘aspirational advice’ on what holism or integration might look like, and ‘pragmatic and context-specific accounts of what worked’ (2024: 4). The Methods section describes the same rationale and context for this review. The Results section situates the search for advice in the context of conceptual ambiguity: what do ‘whole-of-government’ and ‘policy integration’ mean, how do they inform policymaking aspirations or advice, what are the gaps between aspiration and reality, and how do they affect coherence?

The Discussion section synthesises research insights to produce actionable lessons, but not a how-to-guide, for *policy integration in the service of policy coherence.* I use a cautionary tale approach to identify five themes. Explain what policy integration means. Identify your rationale, model, and theory of change. Engage with trade-offs between top-down and bottom-up approaches. Explain why the pursuit of integration has advantages over a reasonable alternative. Learn about facilitators from studies of success and barriers from studies of limited progress. These themes help to address limited progress in relation to clarifying aims, connecting them to routine government business, and making sure there is capacity to deliver. If this advice seems obvious, it should prompt us reflect on why it remains essential despite the stated intentions of so many governments. The idea of policy integration in the service of policy coherence has high rhetorical weight but low real-world traction: governments have a limited willingness and ability to integrate.

## Methods

The series aims to produce connected reviews using a consistent method, so the following description draws on Cairney and Toomey (2024, 2025, see also Cairney, 2025b for added details), which adapts Kuckertz and Block’s (2021) guidance.

*Rationale.* I seek advice on how to improve policymaking with reference to whole-of-government and integration approaches, to inform the Horizon Europe project *Healthy Working environments for all Ages: An evidence-driven framework* (WAge). WAge’s Work Package 1 supports the uptake of evidence, which requires us to understand how evidence-informed collaborative policy processes work, and how they contribute to policy coherence (an integrated ‘healthy working environment’ strategy involves policy responsibilities spread across political systems).

*Engagement with previous reviews.* Cairney leads a team conducting qualitative systematic reviews that connect key aims to policy theory insights ( Cairney *et al.*, 2021a, 2023; Cairney & Kippin, 2022; Cairney & Toomey, 2024, 2025).

*Research/guiding questions.* My general guiding question was: What advice do scholars and practitioners offer on policy integration and policy coherence? I used sub-questions to guide analysis:

1. What terms do authors use to describe their analysis?
2. How do they describe their aims?
3. What factors do they describe as constraining or facilitating integration and coherence?
4. In what context does this pursuit of integration and coherence take place?
5. What findings or recommendations do they provide?
6. To what extent are these lessons transferable to other contexts?

I anticipated that each text would define integration to some extent then offer at least one of three kinds of advice:

1. Aspirational: what would good policymaking look like?
2. General: what context-free advice do people give to foster integration or coherence?
3. Context-specific: what works, for whom, and in what context, and what lessons are transferable?

*Databases and initial search terms.* I use databases produced by Aoki *et al.* (2024a, 2024b), Trein *et al.* (2023) and Trein (2022). Kristin Nicolson downloaded the articles and produced a consolidated bibliography. Aoki *et al.* (2024a) searched Scopus and Web of Science for ‘whole-of-government’ and ‘joined-up government’. Trein *et al.* (2023: 4–5) searched Web of Science (2010–21) for: “Policy integration” OR “Comprehensive planning” OR “Policy coherence” OR “Holistic government” OR “Joined-up government” OR “Whole of government” OR “Horizontal governance” OR “Holistic governance” OR “Policy mainstreaming” OR “Boundary spanning policy regime”. I used snowballing to identify 54 academic and 11 grey texts described by researchers as underpinning their work.

*Timeliness.* Aoki *et al.* (2024a) searched from 1992–2021. Trein *et al.* (2023) searched from 2010–2021 (July).

*Manual searches and choices regarding initial inclusion.* Both teams prioritised academic journal articles in English, and included WG/JUG articles, but have different criteria: Trein *et al.* (2023) searched for more concepts over less time, and included only empirical studies. For Aoki *et al.* (2024a: 736) ‘the exact phrase of WG and or JUG had to feature at least once in the article, excluding references, at the outset. The article also had to elaborate on or exemplify these concepts … for at least one paragraph in the main body of the text’. Trein *et al.* (2023: 5) relate inclusion to: (1) ‘the extent to which they deal with the phenomenon of policy integration (by explicitly referring to policy integration or synonyms of it)’, then (2) ‘articles contained papers that clearly operationalize and examine policy integration empirically’.

**Figure 1.**
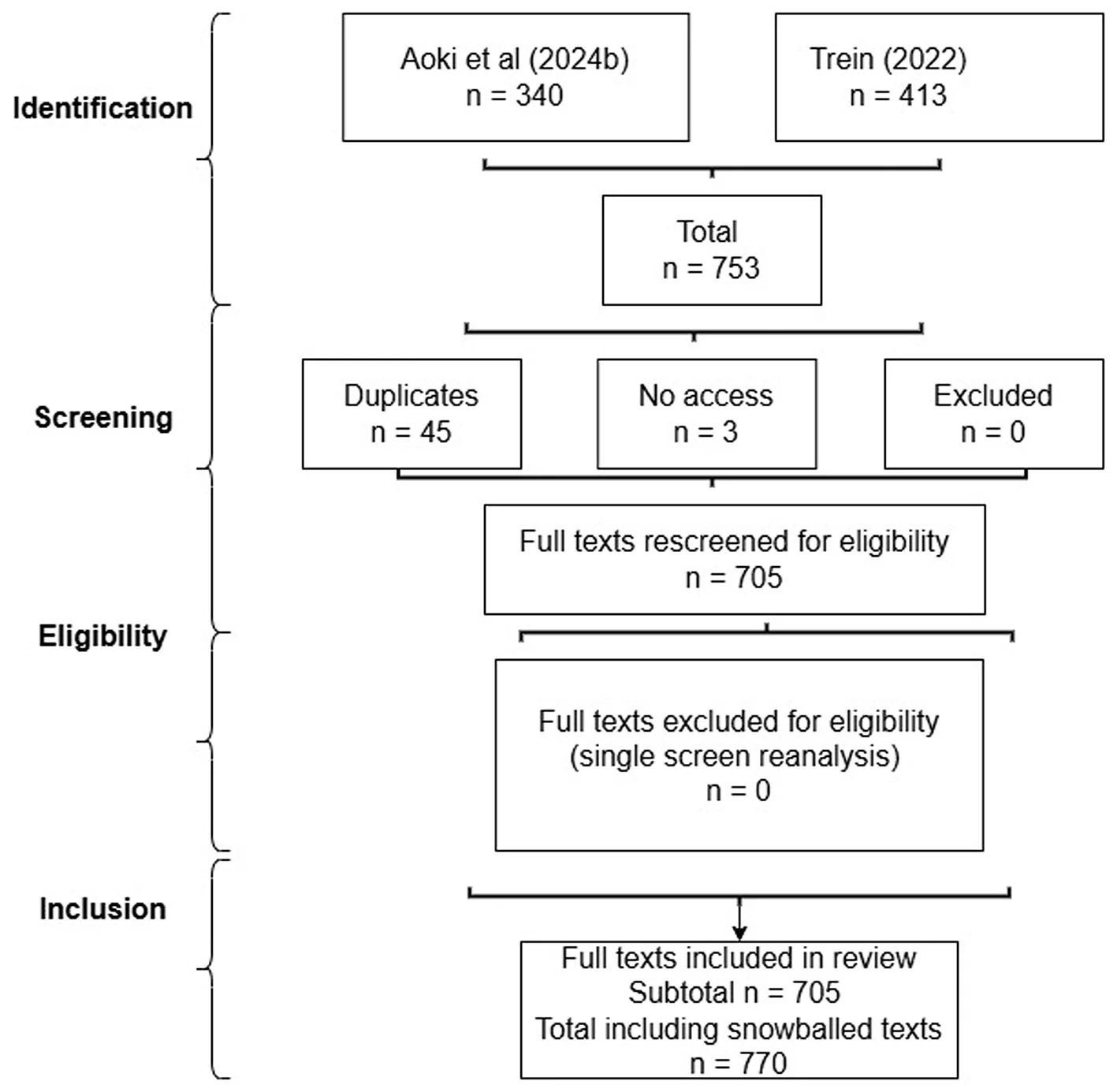
Flow diagram (reanalysis).

*Search results and initial screening* ( Table 1, Figure 1). Aoki *et al.* (2024a: 737) report: 517 texts relevant for screening, 9 not found, 39 wrongly classified, 129 not relevant, leaving 340 (259 WG, 140 JUG, 59 overlaps). Trein *et al.* (2023: 5) report: 1082 texts relevant for screening, 423 eligible, 10 not found (and I identified a database duplicate), leaving 412. There were 44 duplicates/overlaps between both reviews, and 3 no access (Subtotal 705). I did not introduce additional inclusion requirements, but my Results narrative only includes key contributions to my findings ( Cairney, 2025b has a fuller 36000-word account).

**Table 1.** Modified search results 2024–25

Source	Search results	Duplicates	No access	Excluded	Included
Aoki *et al.* (2024b)	340	44 (with Trein)	3	0	293
Trein (2022)	413	1 (database error)	-	0	412
*Subtotal*	*753*	*45*	*3*	*0*	*705*
Snowballing	65	-	-	-	65
Grand total	818	45	3	0	770

*Routine data collection.* Aoki *et al.* (2024a) describe:

• *Conceptual trends.* The number of WG articles has risen from the early 2000s. JUG became static from 2005, following reduced usage by the UK government (2024a: 738).
• *Country/region of study.* Seven ‘leading countries’ – ‘Australia, Canada, the Netherlands, New Zealand, Norway, the United Kingdom, and the United States’ – are the most studied, with UK scholars more likely to study JUG (2024a: 738). Of 235 WG articles specifying a country: 97 were of Australia, 25 US, 22 UK, 19 Canada, 16 New Zealand, Norway, 7 Singapore, 6 South Africa, Netherlands, 5 Japan, Sweden, 4 EU, Germany, 3 China, India, Ireland, Vanuatu, plus 42 single country studies (2024a: 739). Of the 140 JUG: 68 UK, 29 Australia, 11 New Zealand, 9 Norway, 5 Canada, US, Netherlands, 3 South Africa, 17 others (2024a: 740). Australia (126) and UK (90) command 216 (64%) of all studies.
• *Sectors or issues.* Of 317 codable articles: 79 covered health (plus 9 on COVID-19, 2020–21), 43 public order/safety, 40 defence/security/military aid, 40 social protection/welfare, 25 housing/community development, 24 accounting, 24 digital transformation, 22 environment, 11 education.
• *Type of activity.* Almost all focus on coordination across government departments/agencies or sectors (2024a: 739–41).
• *Methods or approaches.* 121 used ‘primary data’, plus 111 interviews, 37 quantitative or mixed methods, 32 surveys, 31 content analysis, 17 ethnographic, 4 ‘regressions’ (2024a: 744)

Trein *et al.* (2023: 8–12, 2019) add the following:

• *Methods.* Most are qualitative (83%, and of a single country 85%). Few are quantitative, but with an upward trend in ‘regression analysis’, networking analysis’, qualitative comparative analysis’ (QCA) and ‘quantitative text analysis’ (2023: 13).
• *Frequency of term usage.* ‘Policy integration’ (61%) and ‘policy coherence’ (41%) are used most, followed by ‘policy coordination’ (20%), WG (18%) and JUG (6%), ‘comprehensive planning’ (urban studies) (7%), ‘horizontal governance’ (3%), ‘policy mainstreaming’ (2%), ‘holistic governance’ (1%). PI is replacing WG as the term for empirical research (2023: 9).
• *Problem focus.* WG studies focus more on organisational coordination and (re) distributive policies (requiring coordination across organisations). PI studies focus on ‘the policy dimension’ and regulation ( Trein *et al.*, 2019: 340–1)
• *Country focus.* PI studies focus more on the EU and continental Europe and less on ‘Anglo Saxon’ studies (both focus similarly on Nordic countries) (2019: 341).
• *‘Stages’ and instruments of policymaking.* Few refer to agenda setting (12%), policy formulation (16%), policy implementation (26%), policy evaluation (6%). More describe policy instruments and mixes (33%, 5%), goals (32%), or design (14%) ( Trein *et al.*, 2023: 10).
• *Policy theories.* Few relate PI to policy theories (e.g. 2% to advocacy coalition framework and multiple streams framework, 1% to punctuated equilibrium theory). More describe policy entrepreneurs (7%) and frames (6%) (2023: 11).

*Data analysis, aggregation, and presentation.* I use an inductive qualitative approach to analyse each text, generate themes manually (Results), and synthesise advice (Results and Discussion) as part of a narrative review (with no numerical qualitative coding). As each review notes, ‘the rules associated with this method are less prescriptive than with its quantitative equivalent, suggesting that we (a) describe each key judgement, and (b) foster respect for each authors methods and aims’ ( Cairney & Kippin, 2022, citing Sandelowski & Barroso, 2007: xv).

There is limited space to synthesise insights from 770 studies, which I addressed initially by producing a 36,000 word account (not including bibliography) ( Cairney, 2025b). Even so, I drew sparingly on 350-400 texts which did little more than express the need for a WG or integrated approach to a policy problem ( Cairney, 2025b notes their applications largely to health, environmental, and foreign and defence sectors). I also synthesized insights from 55 texts which provided a wide range of procedural and analytical requirements for policy coherence, usually without connecting their aims to other sources or a wider literature. I consolidated their insights and requirements to construct the concept of ‘perfect policy coherence’ and prompt debate on researcher aims for coherence (in Table 4, but also published separately as Cairney, 2025c). Most of the remaining articles use many different terms to describe the WG or PI efforts of governments and very few provide explicit policy process advice for integrators. Still, many provided indirect advice by criticizing aspects of the process (such as to caution against too-centralised or top-down approaches) or highlighting the need to account for inevitable barriers to progress. Hence, in the Results section, I try to make sense of the field by categorising PI terms ( Table 2), and the stated purpose of PI ( Table 3), then highlighting debates about the purpose of PI ( Table 5) to provide context for studies of the facilitators and barriers to PI ( Tables 6 and 7), while using considerable space to compare multiple key examples in the literature. Throughout, I draw on a broad reference to mainstream policy process theory insights, on bounded rationality and policymaking complexity, to frame my analysis ( Simon, 1976; Cairney, 2020).

### Limitations relevant to data gathering and analysis

Two previous reviews note five key limitations regarding collaborative policymaking research: the minimal definition of key terms, an umbrella term covers many activities, a limited focus on advice, a dispiriting search for grey literature, and a lack of knowledge (in aspirational research) about how policy processes work ( Cairney & Toomey, 2024, 2025). Our wider suite of reviews also notes that the use of English-language texts exacerbates a bias towards Global North authorship and country coverage. Here, Aoki *et al.* (2024a) and Trein *et al.* (2023) confirm this skewing of included texts to Western and European contexts. Further, while their reviews aid terminological coherence, most of their included studies do not, and most engage sparingly with policy theories or discuss obstacles to progress without advising how to respond. I respond by: keeping the bar for inclusion low to encourage immersion in the field; snowballing to identify key reference points (plus texts from expert recommendations); and, making it explicit that I present advice based on my synthesis of insights.

My search protocol, PRISMA checklist, structured bibliography, and longer draft ( Cairney, 2025b) is stored on Open Science Framework (OSF) https://osf.io/hrbm2/.

## Results

The policy integration literature describes an aim that might seem universally appealing: we need better forms of coordination to pursue a coherent policy mix. Yet, its appeal results from unresolved ambiguity in research and policy when there are too many vague aims and rhetorical commitments. Resolving ambiguity requires researchers and policymakers to explain what integration and coherence would mean in practice, then face trade-offs between competing models of top-down or bottom-up integration. Further, when we examine real world policymaking, we find routine barriers to the vague forms of integration envisaged by reformers. Engaging with these issues of unresolved ambiguity and systemic barriers to integration is the first step to synthesizing feasible advice to guide real world action. To that end, I divide the results into a six-part structure to identify:

1. *The wide variety of concepts and definitions.* The umbrella term ‘policy integration’ shelters many approaches and combines a focus on policy process and outputs.
2. *The large number of general aspirations for change.* Most studies describe vague aspirations, while some contribute to generic advice on how to act.
3. *The need for conceptual clarity: what exactly does integration mean*? There are different approaches and expectations for top-down/centralised or bottom-up/decentralised, and formal or informal integration, across supranational, national, or local scales.
4. *What exactly is policy coherence?* The literature contributes to a remarkably long list of requirements for coherence, describing not only the policy mix but also the processed to produce policy instruments.
5. *What is required of policy integration?* This section summarises more explicit or instructive advice on how to facilitate integration.
6. *The barriers to progress.* Empirical research identifies a routine gap between rhetoric and reality, when non-integration and incoherence are features of policymaking systems rather than bugs.

### The wide variety of concepts and definitions: a clear definition of policy integration is elusive

The study and practice of integration and coherence lacks integration and coherence, partly to reflect incentives in government and academia to reinvent terms or use aspirational slogans ( Cairney & Toomey, 2025; Tosun & Lang, 2017: 554). The umbrella term ‘policy integration’ shelters many variations on a vague theme with no clear origin story. I highlight a full range of concepts in Table 2 then separate two key elements: (1) the pursuit of policymaking integration or integrated policy processes, and (2) the pursuit of policy coherence or a coherent policy mix.

**Table 2.** Variations on the theme of policy integration and policy coherence.

Concept	Summary of aims	Example or citation
*Policy coherence*	Ensure that all relevant policies contribute to a well-coordinated set of aims. Collaborate across sectors to improve the coherence of policy instruments	OECD and UN Sustainable Development Goals (SDGs)
*Whole-of-government approaches*	Coordinate government action and public service delivery towards a shared goal	Population health or climate change policies
*Joined-up government (and holistic government)*	Coordinate government and public sector functions to produce coherent and efficient responses to a problem (e.g. new incentives, departments, or units)	UK Labour government
*Policy integration*	Be aware of the effect of policy in one domain on another, and integrate aims and instruments	Environmental or climate policy integration
*Comprehensive planning*	Centralise the coordination of policy goals or instruments to support a unifying aim	Urban planning
*Strategic state*	Create a country-wide vision, informing aims and objectives, backed by organisations and policy instruments	OECD reports on country government performance ( Elliott & Roberts, 2024)
*Mainstreaming*	Address this aim or problem in all policies	Gender mainstreaming
*Multi- or inter-sectoral action or governance*	Collaboration across policy sectors (inside and outside of government) to share responsibilities for cross-cutting aims	Health in All Policies (HiAP)
*Holistic governance*	Start with clear aims then identify the organisations and instruments to support them	Research and policy agendas for government and public sector reform (e.g. 6, 1997; 6, 2004)
*Horizontal governance*	Coordinate policy and delivery among governmental and non-governmental organisations, emphasising collaboration and shared responsibility	Policies harnessing the involvement of non- government stakeholders (e.g. Ferguson, 2009)
*Network governance*	A decentralised or pluralist way to coordinate policy across governmental and autonomous/ non-governmental organisations. Models may include self-organisation, one leader, or a dedicated administrative structure ( Provan & Kenis, 2008)	(1) Academic research, informing (2) how governance through networks may be effective ( Lewis, 2011a; Sørensen & Torfing, 2007)
*Collaborative governance*	Policymaking ‘across the boundaries of public agencies, levels of government, and/or the public, private and civic spheres’ in relation to a common aim	( Emerson *et al.*, 2012: 2)
*Polycentric governance*	Many policymaking ‘centres’ have high autonomy to act independently, but are motivated to engage in collective action (including to make and follow rules)	Inspired by Ostrom and Ostrom’s ‘Bloomington school’ ( Aligica & Tarko, 2012; Cairney *et al.*, 2019; Jordan *et al.*, 2015)
*Boundary-spanning policy regime (or functional regulatory space)*	Research about the integration of policies and dynamics of subsystems (across multiple policymaking scales or levels). See also Morin and Orsini (2014) on ‘regime complexes’ (networks of international regimes)	Academic research (e.g. Jochim & May, 2010; Varone *et al.*, 2013, Trein, 2017).
*Policy coordination*	Examine if decisions associated with policy programmes or organisations contribute to the same strategy (positive) or do not conflict with others (negative)	Academic research (e.g. Peters, 2015; Peters, 2018; Russel *et al.*, 2020: 5)
*Policy mix coherence*	Research on the extent to which multiple policy instruments combine to complement or contradict each other	‘New policy design’ (e.g. Howlett, 2014; Peters *et al.*, 2018; see also Simoes *et al.*, 2015)

Source: author’s own from Tosun and Lang (2017); Trein et al. (2023); Aoki et al. (2024a) unless stated.

This analytical distinction between policy processes and outputs reflects existing distinctions in research between a policy theory focus on *policy processes* and research and advice on *policy analysis* ( Cairney, 2020, 2021a; Peters *et al.*, 2018; Trein & Maggetti, 2020: 199; Trein *et al.*, 2021: 1411; Weible, 2023). These literatures may inform different searches for advice or frame that advice in different ways.

*Policy processes*: *to make and influence policy, to turn beliefs or aims into policy.* The *practical aim* of policymaking integration is to transform policy processes to oblige integration or foster voluntary collaboration among multiple actors. Collaboration can be designed to transcend policy sectors, government departments or agencies, or inside/outside of government. Collaboration can boost ‘policy capacity’, or the ability of many organisations, or networks of public and private actors, to harness resources ( Williams & McNutt, 2013).

The *research aim* of policy theories is to compare these intentions with real-world practices and identify the causes of gaps ( Cairney & Toomey, 2024, 2025). For example, our review of Health in All Policies (HiAP) identified strong reasons for specialisation in ‘subsystems’:

“classic studies of ‘policy communities’ highlight a logic of delegating policy responsibility to junior civil servants, engaged in routine consultation with a limited number of actors who trade information and advice for access … Most policy is processed in silos that seem to defy central coordination. Silos develop rules appropriate to their own contexts, and their logics do not change simply because the overall effect looks like uncoordinated and incoherent policymaking … [while some describe] these dynamics as obstacles to be overcome, policy studies present them as ever-present forces to which to adapt. A lack of intersectoral action seems incoherent to some but *makes sense* to others” ( Cairney *et al.*, 2021a: 24–5; see also Cejudo & Trein, 2023; Jochim & May, 2010: 304; Peters, 2015; Peters, 2018: 4–5; Tosun & Lang, 2017: 554).

This argument that *silos and subsystems make sense* reflects positive and negative drivers including:

• the benefits of specialization such as expertise and collaboration between a small number of actors
• the benefits of limiting attention and simplify tasks
• to connect accountability to specific actors (not collaboratives)
• the politics of policymaking, including messy compromises between parties ( Bolleyer, 2011; Bressanelli, 2013) and turf wars between departments ( Arbeiter *et al.*, 2019: 466–7; Daviter, 2017: 579–81; Peters, 2015; Peters, 2018: 4–5; Tosun & Lang, 2017: 553; Verhoest *et al.*, 2007).

This argument also cautions researchers to see fragmentation as evergreen: ‘Policy integration is … not a single moment when those tensions are solved … but a political process that requires deliberate efforts to overcome the pull toward sector-specific’ dynamics ( Cejudo & Trein, 2023: 9).

*Policy outputs*: *the instruments to influence individual, social, or organisational action to change policy outcomes.* The *practical aim* is to pursue policy coherence, such as to design and deliver a coherent policy mix. This mix can consist of policy instruments to redistribute or distribute resources, regulate social or market behaviour, build organisational capacity, and/or gather evidence and share information. It may be produced by organisations spread across multiple scales or levels of policymaking. Each instrument may have different ‘targets’ and ‘beneficiaries’ ( Flanagan *et al.*, 2011: 707; Hood & Margetts, 2007; Howlett, 2014; Rogge & Reichardt, 2016).

The *research aim* is to explain why individual instruments have limited effects, or why the mix is incoherent ( Howlett *et al.*, 2015; Peters *et al.*, 2018). Studies find an unclear connection between design processes and instruments ( Howlett, 2014; Peters *et al.*, 2018). This disconnect is multiplied when many policymaking organisations are spread within or across policy sectors and levels of government, and have some powers to produce some instruments for different purposes and make or deliver policy at different times, thus prompting an overall approach and policy mix that is difficult to coordinate and cohere ( Bahn-Walkowiak & Wilts, 2017: 165; Biesbroek & Candel, 2020: 64; Cairney, 2021a; Cairney *et al.*, 2022a; Candel & Biesbroek, 2016; Howlett *et al.*, 2017: 72; May *et al.*, 2006: 383; Righettini & Lizzi, 2020; Sandström *et al.*, 2020; Simoes *et al.*, 2015).

This simple analytical distinction between policy processes (policymaking integration) and outputs (policy coherence) is difficult to maintain but provides a useful lens through which to view each major approach to PI.

***Whole-of-government and joined-up government approaches***

WG and JUG are vague terms ( Christensen & Lægreid, 2007) and Aoki *et al.* (2024a: 736) treat them as ‘interchangeable’. Descriptions of JUG could be of WG, including ‘the aspiration to achieve horizontally and vertically co-ordinated thinking and action’ ( Pollitt, 2003: 35 in Davies, 2009: 81; Hodges, 2012: 30; Karré *et al.*, 2013: 63; Wiggan, 2007: 415). Here, horizontal may describe coordination across the same level of government, while vertical may describe coordination across multiple levels (but it is always worth checking what the authors mean).

To some, WG represents an attempt to shift away from New Public Management (NPM). Its focus on ‘structural devolution, disaggregation, and single-purpose organizations’ produced a fragmented government and public sector, which left it ill-placed to address the ‘wicked’ policy problems that transcended sectoral and organisational responsibilities ( Christensen & Lægreid, 2007: 1059, citing OECD, 2005; see also Exworthy & Powell, 2004; Flinders, 2002: 53; Halligan *et al.*, 2011: 78; Kraak, 2011: 345–8). Central governments who had embraced NPM – especially in the UK, New Zealand, and Australia – sought to deal with the unintended consequences: they had sought to delegate responsibilities and share accountability but lost central control *and* faced the blame for poor outcomes (2007: 1060; Verhoest *et al.*, 2007: 326; see Flinders, 2002: 57–60; Heppell, 2011; Hodges, 2012; Kavanagh & Richards, 2001: 3–8; Lewis, 2011b on the UK).

There are country-level variations to reflect scale, political system, and the chosen balance between centralisation and local autonomy and collaboration ( Halligan *et al.*, 2011: 77; Ling, 2002: 619–21). Still, commonalities include to foster integration by treating citizens or service users as customers, perhaps as part of a ‘one-stop-shop’ ( Hammerschmid *et al.*, 2019). Further, while narratives of policy problem ‘wickedness’ are vague ( Daviter, 2017: 575), they are strong enough to convince governments to reassert control over their response to multiple existential security, environmental, and other crises (2007: 1060) and to address the lack of clarity regarding formal responsibilities (e.g. Ison *et al.*, 2018; Yusuf *et al.*, 2022: 487; Young & Grant, 2015: 16).

***Policy integration***

Tosun and Lang (2017: 553) use ‘policy integration’ as the umbrella term to describe (1) ‘the collaboration of actors from two or more policy domains’ to (2) ‘integrate aims and concerns derived from one policy domain into another’. Cejudo and Trein (2023: 9) also describe the political activity and intended output:

‘Policy integration is a political process that entails the coordination of actors and agencies across policy subsystems, the combination of instruments from different policy sectors, as well as arrangements for their consistent implementation and evaluation, as a response to a complex policy problem that not one policy sector, policy instrument, or agency can solve.’

Rayner and Howlett’s (2009: 99–100) definition of ‘integrated policy design’ focuses on the outputs and outcomes:

- “Integration is the replacement of specific elements of existing policy ‘mixes’ or ‘regimes’ – the goals, objectives and calibrations of existing policy tools and goals – by a new policy mix, in the expectation of avoiding the counterproductive or sub-optimal policy outcomes that arise from treating interrelated policy regimes and components in isolation from one another”.

Environmental and climate policy accounts for much research, reflecting strong rhetorical commitments by governments and ‘international governmental organisations’ (IGOs) to environmental policy integration (EPI) and growing commitment to climate policy integration (CPI) ( Cejudo & Trein, 2023: 11; Tosun & Peters, 2018a; Tosun & Peters, 2018b: 90). While ‘integration’ is vague, approaches can signal high ambitions. For example, *integrated policy strategies* (IPS) describes the pursuit of ‘a set of integrated goals by a polity as a whole’ which may involve a new ‘complex multilevel’ policy mix to reflect its importance across policy sectors and levels of government, backed by ‘legislation, programmes and/or high-level agreements’ ( Candel, 2017: 520). Examples include IPS for ‘food insecurity, climate change, or homeland security’ (2017: 520). Here, policy actors attempt ‘to re-design entire policy regimes around new policy paradigms, such as sustainability’ ( Rayner & Howlett, 2009: 100). See also ‘strategic state’ approaches regarding the ‘creation and delivery of an effective strategy at a country-wide level’ ( Elliott, 2020: 286; Elliott & Roberts, 2024: 5; Joyce, 2022; Joyce & Drumaux, 2014; OECD, 2013: 58).

***Mainstreaming***

Mainstreaming is to consider one problem or aim in all policy. Examples include environmental, climate adaptation, or ‘ecosystem services’ mainstreaming ( Nunan *et al.*, 2012; Rauken *et al.*, 2015; Rosenberg, 2020; Schleyer *et al.*, 2015; Schubert *et al.*, 2018; Wamsler *et al.*, 2014; Wamsler & Pauleit, 2016; Widmer, 2018) and HiAP ( Cairney *et al.*, 2021a; Peters *et al.*, 2016; Ramirez-Rubio *et al.*, 2019). ‘Policy coherence for development’ (PCD) or ‘sustainable development’ (PCSD) ( OECD, 2023) seeks to mainstream economically developing country concerns in developed country policies ( Adelle & Jordan, 2014; Carbone & Keijzer, 2016; Fritz & Raza, 2017; Janský, 2015; Mbanda & Fourie, 2020; Nshimbi, 2019; Prontera, 2016: 299; Verschaeve *et al.*, 2016; Zeigermann & Böcher, 2020).

Mainstreaming may also reflect the need to address the gendered or racialised division of power that marginalises some social groups from meaningful partnerships or collaborative efforts ( Scott & Thurston, 2004). For example, ‘gender mainstreaming’ (GM) may begin strongly, such as when the Council of Europe (1998: 15) defined it as ‘the (re) organization, improvement, development, and evaluation of policy processes, so that a gender equality perspective is incorporated in all policies at all levels at all stages, by actors normally involved in policy making’. However, the means to achieve GM vary markedly: some governments exhort or set benchmarks, while others produce substantive regulatory or redistributive policy instruments. This responsibility is shared across levels, often causing policy dilution ( Cairney *et al.*, 2021b, 2022a: 147; Pollack & Hafner-Burton, 2010: 293, citing e.g. Abbott & Snidal, 2000; Beveridge, 2012; Daly, 2005; MacRae, 2010; Peck, 2002: 332; see also the new review by St. Denny & Cairney, 2026).

### Vague aspirations for whole-of-government approaches and integration

Cairney (2025b) summarises the large number of studies (most of the 705 texts) that contribute to at least one of four statements:

1. I recommend a whole-of-government or integrated approach to this wicked policy problem.
2. Here is what a whole-of-government or integrated approach would look like.
3. This government describes an aspiration to take a whole-of-government or integrated approach, but in a vague way.
4. This government has used a WG approach successfully, but it is difficult to connect its success to WG factors.

Most of this literature uses the general PI lexicon to exhort a holistic understanding of a wicked problem, encourage more coordination, then find limited progress, and push for change. Cairney (2025b) summarises hopes for WG approaches to:

• Public health. Health protection, health improvement, mainstreaming public health in foreign and domestic policy, boosting citizen participation in health governance, a One Health approach (connecting humans, animals, environments), and integrating public services.
• Sustainability, environmental protection, climate mitigation and adaptation, and disaster preparedness. Sustainable development, a ‘nexus’ between energy, land, water, and natural resources, plus conservation and biodiversity management, and disaster management or mitigation.
• National security. Integrate domestic and international affairs, including foreign policy, security, terrorism, defence, peacebuilding, diplomacy, and aid policies.
• Governance issues, including the patchy and often unsuccessful use of information and communication technology (ICT), development of Whole of Government Accounts (WGA), and efforts to collaborate with indigenous communities.

Such studies identify general ‘enablers’ including capacity, communication, collaborative governance, leadership, evidence generation, backed by accountability measures (e.g. Agrawal *et al.*, 2021: S28; Alexander & Haward, 2019: 35–6; Cavallo *et al.*, 2017; Cavallo *et al.*, 2018; Gerhardinger *et al.*, 2019; Hassler *et al.*, 2018; Mascarenhas *et al.*, 2015; Mongruel *et al.*, 2013; Morf *et al.*, 2019: 206–8; Nalau *et al.*, 2016: 372; Orderud & Naustdalslid, 2020: 25; Sander, 2018; Schubert *et al.*, 2018: 308; Wurtzebach *et al.*, 2019: 130).

There are some success stories in country studies of COVID-19, citing factors such as: clarity of purpose, effective leadership and collaboration ( Hsieh *et al.*, 2021: 310–12), ‘collaborative innovation’ to bolster investment in ICT capabilities ( Lee *et al.* (2023: 340); ‘collaboration from non-government organisations and private sectors’ ( Ang *et al.*, 2021); and, ‘strong resource mobilization and control of government departments, companies, and citizen communities’ ( Jing & Xue, 2021: 31). However, it is difficult to identify (1) a clear link between identified WG mechanisms and the successful outputs described in these accounts (although Hsieh *et al.*, 2021 and Lee *et al.*, 2023 reflect on preparedness after a previous SARS outbreak), or (2) how these approaches differed from the countries who used similar WG measures (e.g. the UK – Cairney, 2021b) (see also the new review of COVID-19 policy by Cairney & Intropido, 2025).

### The need for conceptual clarity: what exactly does policy integration mean?

Most PI studies produce too-general aspirations with an unclear connection to action. This field would be more conducive to coordinated growth if informed by the following guiding questions summarised in Table 3.

**Table 3.** What is the point of policy integration?

*Terminology*	Whole-of government, joined up government Policy integration
*Sincerity*	To do something To look like you are doing something
*Origin story*	Seek central government control in response to crisis Assert national central government control in a multi-level system Address the unintended consequences of previous reforms Address a wicked policy problem more effectively Improve democracy to improve policymaking
*Priority*	Coordinate policies from international to local action Remove barriers to cooperation across sectors, departments, silos Produce one plan for government in a country Foster collaboration across public services Make policy in collaboration with stakeholders and citizens
*Level*	One level of government, focused on horizontal coordination Multiple levels, focused on vertical integration (e.g. central-local)
*Scale of inclusion*	Policymakers across government departments Actors who make and deliver policy across the public sector Actors inside and outside of government
*Method, means, strength*	Centralised and top-down or decentralised and bottom-up Hierarchy, market, or network mechanisms Hard or soft measures
*Intensity of coordination*	More information sharing by many autonomous actors One strategy enforced on all actors One unit to make and deliver all policy A ‘one stop shop’ to coordinate all relevant public services
*Policy problem*	Wicked or tame issues Requires an ambitious policy mix, produced by many sectors Requires one overarching strategy and regulatory framework
*Timeframe*	Rapid reform for immediate impact Anticipation, prevention, planning for the long-term
*Measure of progress*	The reform is popular The process enjoys legitimacy and ownership There is evidence that the policy works as intended when implemented

**Terminology: what terms are used and what do they mean?**

Governments and advocates describe WG vaguely, without clarifying what it would look like if successful. In research, PI is broad and varying usage creates confusion ( Trein *et al.*, 2023: 3–4). If PI can describe so many activities ( Table 3), actors should avoid using it loosely.

**Sincerity: what is the level of commitment to reform?**

Serious reformers seek a meaningful shift in thinking and behaviour ( Keast, 2011: 230; Molenveld *et al.*, 2021: 124). Others use ambiguity strategically, using ‘buzzwords’ to pretend to act ( Christensen & Lægreid, 2007: 1061, citing Gregory, 2006). Given this range of possibilities, PI means little unless backed by a means to translate ambition into deliverable instruments.

**Origin story: what is the main motivation for integration?**

We require a clear stated rationale when the field contains many motivations. First, *to assert power or control.* It includes to reassert central government power when addressing crises or managing coalition politics ( Halligan *et al.*, 2011: 79–80; Verhoest *et al.*, 2007: 341). Or, to reassert *national* central government in multi-level governance arrangements where responsibilities may be shared ( Trein & Maggetti, 2020: 20; Trein *et al.*, 2021).

Second, *to respond to the unintended consequences of previous reforms.* NPM exacerbated silos and fragmentation and undermined central coordination ( James, 2001; Tosun & Lang, 2017: 557).

Third, *to address a ‘wicked’ policy problem more effectively* ( Verhoest *et al.*, 2007: 341; Tosun & Lang, 2017: 561–46, 1997). To avoid the costs of a lack of coordination, including: ‘ *duplications’* of effort; ‘ *contradictions’* between instruments with their own rationales; ‘ *displacement’* of work; too much focus on ‘ *vertical management’* within a hierarchy rather than horizonal cooperation; and, a lack of response to ‘ *changing demands’* or ‘ *cross-cutting problems’* ( Peters, 2018: 4). Further, some left-wing parties are more interested in joining-up state intervention for HiAP, environmental justice, or social investment ( Trein & Ansell, 2021: 1148–9).

Fourth, *to seek the benefits of improving participatory or deliberative democracy.* The inclusion of citizens and stakeholders can boost policymaking knowledge and policy ownership ( Tosun & Lang, 2017: 563; e.g. Franzén *et al.*, 2011; Milhorance & Bursztyn, 2018; Mullally *et al.*, 2018; Nadin *et al.*, 2021: 794–5; Zhang *et al.*, 2020).

Or, the origin story may be holistic, such as to describe the NPM legacy and inability of fragmented governments to address wicked problems, then present a vision for more anticipatory and preventive policymaking that fosters trust-based collaboration and empowers public sector workers to meet the needs of their population (6, 1997; 6 *et al.*, 2002).

**Priority: what is the main aim of integration?**

The aim may be enduring coherence when policy is produced and delivered by multiple countries and international organisations ( Tosun & Lang, 2017: 556–7; citing OECD, 2009; see also Desta & McMahon, 2015; Hezri & Dovers, 2009; Koff *et al.*, 2020; Le Blanc, 2015; Mohammadi *et al.*, 2019; OECD, 2019, 2023, 2024; Ruffing, 2010; Wong, 2019a). WG focuses on issues like government departmental silos to be knocked-down or joined-up. Terms like ‘comprehensive planning’ described centralised coordination to manage ‘multiple goals in urban planning’ (2017: 556; Jun, 2017; Jun & Conroy, 2013; Jun & Conroy, 2014; Trein *et al.*, 2023: 8). ‘Strategic state’ extends the scale of planning to a country’s government ( Elliott & Roberts, 2024). Other terms focus more on governance, to foster less hierarchical coordination of public service management or policy delivery, or to prioritise stakeholder or citizen engagement ( Tosun & Lang, 2017: 556–61). This framing of priorities matters whenever broad aims cohere in theory but collide in practice.

**Scale of inclusion: which actors or organisations are essential to coordination?**

Initiatives may envisage a core group of actors coordinating across a central government, a larger group facilitating action across the entire public sector, or participation on a grander scale and inside and outside government.

**Level: how many, and what levels of government are involved?**

Initiatives can promote horizontal and/or vertical coordination. One WG response to NPM was to refocus on cooperation across government departments (horizontal) and address problems with the performance management of local service delivery (vertical) ( Christensen & Lægreid, 2007: 1060).

**Method: what are the means to ensure integration, and how strong are they?**

Integration can be driven by one central government or decentralisation and collaboration in relation to delivery and local engagement ( Keast, 2011: 222). Studies distinguish between coercive central government ‘mechanisms’ for ‘hierarchy’, ‘market’ mechanisms such as incentives and internal-markets, and ‘network’ mechanisms to coordinate via ‘consensus, trust, and mutual dependence’ ( Molenveld *et al.*, 2020: 1; Verhoest *et al.*, 2007: 332). Christensen and Lægreid (2007: 1061–62) distinguish between:

• ‘hierarchical-instrumental’, an ‘aggressive top-down style’
• ‘negotiation-instrumental’, a pragmatic search for collaboration
• ‘cultural-institutional’, a more organic ‘evolution’ of public service
• ‘myth-institutional’, or stories of reforms which could be ‘fads’ or ‘window dressing’.

‘Top-down’ can be used negatively to warn against unintended consequences or normative problems ( Christensen & Lægreid, 2007: 1061, citing Richards & Smith, 2006). Or, positive usage describes much-needed direction (e.g., Sander, 2018). The latter informs the distinction between ‘hard’ and ‘soft’ measures of substance:

• ‘Hard’ describes ‘three dimensions of obligation, precision, and delegation, with (1) binding provisions entailing (2) precise responsibilities and commitments, backed by (3) strictly enforced positive and negative sanctions for compliance and noncompliance’.
• ‘Soft’ measures include ‘(a) non-binding provisions with (b) vague or imprecise aims and (c) little or no attempt to monitor and sanction officials for compliance and noncompliance’ ( Pollack & Hafner-Burton, 2010: 292, citing Abbott & Snidal, 2000 on GM; Tosun & Lang, 2017: 562; see also Widmer, 2018: 72). For example, UN SDGs represent ‘the softest of all global governance tools’ ( Wong, 2019a: 2).

**Intensity: what is the level of obligation to integrate or collaborate?**

Metcalfe (1994: 281) describes an ascending scale of policy co-ordination within a country, from independent decisions, to ‘information exchange’, ‘consultation’ and ‘feedback’, ‘avoiding divergences’, ‘search for agreement’, ‘arbitration of policy differences’, ‘setting limits’ on the extent to which one policymaker can diverge, ‘establishing central priorities’, and one ‘government strategy’. This scale is a tool to gauge or encourage horizontal coordination (1994: 287–8; Dafinova, 2021: 466; Flinders, 2002: 61; Wilkins, 2002: 117; Wong, 2019a: 2).

Equivalent efforts towards vertical integration include a push towards ‘shared services’ ( Valkama *et al.*, 2016) or a ‘one stop shop’ to offer citizens a single contact for public services ( Wiggan, 2007; Wilkins, 2002: 115–6). If such coordination relates to non-governmental or semi-autonomous organisations, and the means is unclear, even the strongest ambition has limited impact ( Molenveld *et al.*, 2020: 11). Consequently, there are also measures of cooperation, such as participation at multiple decision points, partnership working, compromise, or other means to boost stakeholder ownership ( Aurich-Beerheide *et al.*, 2015: 382; Ribalaygua Batalla & García Sánchez, 2016, citing Pollitt & Bouckaert, 2011).

**Policy problem: how wicked is the issue, and to what extent does it transcend sectors?**

Richards (2001, in Christensen *et al.*, 2014: 243) distinguishes between ‘wicked problems’ that transcend sectoral and jurisdictional boundaries, ‘tame’ issues conducive to top-down coordination, and ‘seamless services’ aided by ICT. WG cooperation is key when multiple actors are responsible for redistributive and regulatory policies (e.g. HiAP), while PI is more of a feature of regulatory approaches (e.g. climate change agreements) ( Trein *et al.*, 2019: 340). That said, reframing climate *change* to *justice* would prompt attention to a wider suite of instruments, such as to include measures to recognise the role of participants and promote fair processes as well as to monitor unequal outcomes ( Cairney *et al.*, 2023).

**Timeframe: how long will it take to reform and see results?**

Ambitious reforms may have visible short-term effects but uncertain long-term consequences ( Christensen & Lægreid, 2007: 1063). There is no commonly agreed language or metric to describe or gauge such progress ( May *et al.*, 2006).

**Measure of progress: what determines policy success?**

Studies may examine how evidence-informed is the strategy and its evaluation: does advice relate only to aspiration or is it backed by well-documented evaluation? This focus on evaluation and appraisal is political: there is contestation to determine the goals, measures, and evidence for success or failure. Debates juggle three broad criteria to assess success:

• *Political.* Does it boost trust in government or policymaker popularity?
• *Process.* Is it possible to create and maintain legitimacy and support for this policy?
• *Programmatic.* Does this policy achieve its stated goals and outcomes? ( McConnell, 2010; see e.g. Scott & Boyd, 2023)?

If success relates as much to a strategy’s impact on popularity as its socioeconomic impact, we return to the need to establish meaning (is this policy largely rhetorical to stay popular?).

### What exactly is policy coherence? Aspiration and reality

I argue above that PI research describes (and often conflates) two aspects: (1) coordination to support policymaking integration, in the service of (2) policy coherence or a coherent policy mix. Here, I note the same problem of conflation in studies of policy coherence. Policy coherence research began as a broad way to describe a policy mix: ‘various policies go together because they share a set of ideas or objectives’. However, my review identifies an expansion of meanings and ambitions associated with this term: many ‘policy coherence’ studies seek to go further, to describe the need for multiple efforts towards policymaking integration (coordinative practices) and multiple measures of policy mix coherence (see Cairney, 2025b for a full bibliography). The outcome of literature synthesis is a long and potentially confusing shopping list of requirements that could not all be met ( Table 4). Indeed, by producing and analysing this list, we can see that advocates of policy coherence often present very different visions of the processes involved, including relatively top-down and centralist versus bottom-up and decentralist ambitions. I explain this argument more fully in Cairney (2025b) and Cairney (2025c) which uses this list to construct an ideal-type ‘perfect policy coherence’ to compare with policymaking in the real world (drawing on Hogwood & Gunn, 1984 on ‘perfect implementation’). Here, I describe each indicator briefly and identify the competing ideas that emerge.

**Table 4.** Indicators of policy coherence.

Category	Example
Procedures	The policy process is clear, consistent, and predictable Actors know when to get involved or share evidence
Agendas	There is high attention and priority to a policy problem The problem’s definition (framing) is clear and consistent
Analysis	Problem diagnosis is evidence-based Solutions will work as intended if implemented Normative beliefs align with empirical expectations There is a clear and convincing theory of change
Actors	There is substantive involvement among all relevant actors Key actors share responsibility for the problem and ownership of solutions
Consensus seeking	There are effective ways to manage competing beliefs and interests Seek consensus through collaboration, not imposition
Levels of government	There is a means to coordinate a coherent strategy or ensure that strategies do not undermine each other
Instruments	A policy mix contains mutually supportive instruments
Implementation	Delivery resources are in place Delivery does not undermine strategy
Outcomes	The strategy works as intended The policy mix sends clear signals. Recipients experience coherence
Monitoring and evaluation	There are agreed targets. Reaching a target signals substantive progress. There is high agreement on indicators of success
Time	A strategy and mix remains coherent in the short- and long-term
Fit for purpose	The strategy is credible and durable

***Empirical studies seeking policy coherence***

Elements of Table 4 can be used to promote change, such as to:

• establish an ideal to which to aspire ( Ishii & Langhelle, 2011)
• assess a strategy’s credibility ( Nadin *et al.*, 2021)
• measure progress in relation to one element ( Rega & Bonifazi, 2014: 1355)
• improve connections between separate strategies ( Strambo *et al.*, 2015)
• ‘plug gaps’ in old strategy with a new initiative (e.g. Voyer *et al.*, 2020)
• compare coherence in ‘policy discourse and negotiation’ with ‘policy goals and instrument formulation’ and implementation ( von Lüpke & Well, 2020: 841–2)
• track levels of coherence during crisis management ( van der Lijn, 2015: 85–86)
• build on a legacy of collaboration and demonstrable success ( Gössling, 2013: 204; Plowden, 2020: 160; Söderberg, 2011: 532; Wamsler & Pauleit, 2016: 82).

Some approaches seek to combine multiple Table 4 elements to produce a sophisticated empirical model of coherence. For example, Pham-Truffert *et al.* (2020) draw on Nilsson *et al.*’s (2018) ‘seven types of interactions between SDG targets’ to identify which actions create ‘co-benefits without much risk of producing trade-offs’ and which have with the most potential to shift from negative to positive cycles (2020: 1247; see also Kettner & Kletzan-Slamanig, 2020: 144–5; Martin *et al.*, 2014: e391–95; Mickwitz *et al.*, 2009; Niedertscheider *et al.*, 2018: 11; Nykiforuk *et al.*, 2019: 34; Pengilley & Kelly, 2018: 5; Ronzon & Sanjuán, 2020; Rudd *et al.*, 2018: 4; Russel *et al.*, 2018a: 45; Shill *et al.*, 2012: 169; Šumrada *et al.*, 2020; Taylor & McAllister, 2014: 514–15; Zinngrebe, 2018: 155–56).

Some approaches identify the policy instruments essential to policy integration (coordinative or procedural) or a comprehensive mix (substantive). Coordinative instruments include to reform structures and manage collaborations ( Verhoest *et al.*, 2007: 345; see also Antwi-Agyei *et al.*, 2017: 5; Aubrechtová *et al.*, 2020: 4; Baulenas & Sotirov, 2020: 3; Becken *et al.*, 2020: 1611; Benson & Lorenzoni, 2017: 1924; Kelleher *et al.*, 2019; Koff, 2020: 414; Okpara *et al.*, 2018; Oliveira *et al.*, 2019; Reyes-Mendy *et al.*, 2014). Similarly, procedural instruments foster cross-government collaboration and the alignment of multiple subsystems ( Lang, 2019). Examples of substantive policy instruments include the ‘priority interventions’ on non-communicable diseases ( Mwangi *et al.*, 2021: 11), ‘upstream’ interventions to address ‘substance misuse’ ( Vimpani, 2005: 116–20), ‘food security’ coherence via production, provision, nutrition, and resilience to extreme events ( Lowitt *et al.*, 2016), or regulations, standards, and guidance for sustainable cities ( van Stigt *et al.*, 2013: 226).

***Competing perspectives and barriers to policy coherence***

From what or whose perspective should we understand coherence?

*Top-down and bottom-up perspectives.* Table 4 largely produces a top-down view, to imagine one centre of authority seeking to design and deliver a coherent strategy, then trying to close an implementation gap. Multiple studies explore coherence from other perspectives, such as among the local governments or street-level bureaucrats trying to make sense of policy in practice ( Sevä & Sandström, 2017, drawing on Lipsky, 1980; see also Jessen & Tufte, 2014; Røysum, 2013; van Stigt *et al.*, 2013: 231). The normative issue regards who is in charge and if they have sufficient coordinative resources (e.g. Antonson *et al.*, 2016; Berg *et al.*, 2010; Berke *et al.*, 2013: 458; Bjärstig *et al.*, 2018; da Costa Canoquena, 2013; Velázquez Gomar, 2016). The practical or empirical issue regards what happens when top-down expectations for overall strategic coherence (e.g. at the international or national central government level) meet with bottom-up practices that give other levels of government or organisations the discretion to go their own way. Combining these normative and empirical issues, we find not only contestation about how to pursue coherence, but also how to describe it: a top-down perspective focused on vertical PI (integration across levels of government to boost overall coherence), may identify incoherence at local levels, while a bottom-up approach may identify a coherent approach in each local context (see next section on competing approaches to PI).

*Sectoral perspectives.* Similar tensions exist between separate efforts towards integration without an agreed focal point in a place or sector ( Khan *et al.*, 2018). Different actors may perceive ‘their’ issue to be at the heart of integrative efforts, and an issue treated as the main priority for one subsystem may be largely ignored in another.

*Critical perspectives.* Critical scholarship questions the purpose and power dynamics associated with coherence agendas. For example, sustainable development may be a vehicle to address climate change while prioritising economic growth and maintaining the primacy of capitalism, rather than a radically new model of social, economic, and political activity (e.g. Cairney *et al.*, 2023; Kurze & Lenschow, 2018; see also Kivimaa & Virkamäk, 2014; Rauken *et al.*, 2015; Sjöstedt & Kleinschmit, 2016). If so, only measuring coherence misses the bigger picture.

***The coherence gap: vague and contested aims with unclear outcomes***

These competing perspectives throw up the possibility of periods of coherence and incoherence over time, such as if one governmental, sectoral, or procedural approach dominates, followed by bursts of enthusiasm for modifying priorities or approaches (e.g. Di Gregorio *et al.*, 2017; Eadson, 2016; Göpfert *et al.*, 2019; Hommels *et al.*, 2013: 455–6; Mullally *et al.*, 2018: 75; Rouillard *et al.*, 2013). They inform the second main purpose of Table 4: to use each requirement to explain an absence of policy coherence ( Sahin & Feaver, 2013: 1075; Scobie, 2016: 27). Comparing requirement with reality helps to identify problems with:

*Procedures*, such as the absence of a process to resolve multiple sources of incoherence, including internal (competing aims), vertical (competing central-local priorities), horizontal (competing sectoral aims), and transnational (national outcomes do not fulfil international commitments) ( Mallory, 2016).

*Agendas*, when incoherence reflects low priority, attention, or action ( Feola *et al.*, 2019; Füller *et al.*, 2018; Ruckert *et al.*, 2017: 86; Soria *et al.*, 2020).

*Analysis*, including unresolved trade-offs between aims ( Rietig, 2013: 298).

*Actors*, such as when networks do not fully include actors essential to design and implementation ( Locatelli *et al.*, 2020).

*Consensus seeking*, such as tokenistic collaboration ( Satumanatpan & Chuenpagdee, 2015: 16; Schunz *et al.*, 2021; Selianko & Lenschow, 2015; Vasileiadou & Tuinstra, 2013; Zegras & Rayle, 2012: 313). Unresolved conflicts of beliefs lead to policy changes without the participation of some groups ( Sarvašová *et al.*, 2013: S70–71) often ‘to the extent that actors disrespect integration mandated and incentivized by laws’ ( Metz *et al.*, 2020: 8).

*Levels of government*, such as when the ambiguity of a high-level strategy reflects a lack of senior policymaker support, unresolved sectoral or organisational competition, and limited staff resources to translate key aims during local implementation ( Russel *et al.*, 2018a: 48–49).

*Instruments*, when some instruments are more developed and supported than others ( Makkonen *et al.*, 2015: 161; Nadin *et al.*, 2021: 796).

*Implementation*, including ‘barriers’ to progress beyond strategies ( Karlsson-Vinkhuyzen *et al.*, 2018: 134; Miller *et al.*, 2018; Waylen *et al.*, 2019; Zinngrebe, 2018: 157). A major strategy is too (a) vague, superficial, or tokenistic, and (b) disconnected from implementation or delivery, to give any confidence on its likely effect (e.g. Isoaho *et al.*, 2019; Kalaba *et al.*, 2014; Larsen & Powell, 2013; Ranabhat *et al.*, 2018: 975; Rouillard *et al.*, 2013: 384; Steurer & Berger, 2011).

*Outcomes*, when policy instruments do not work as intended when implemented ( Hapuarachchi *et al.*, 2016; Metz & Glaus, 2019: 1).

### What is required for policy integration?

Our review of systems leadership identified training requirements, to develop the mindsets and skills for collaboration and boundary spanning, within a supportive governance architecture ( Cairney & Toomey, 2025). In PI research, there is a more contested account of what is required ( Table 5), reflecting competing ideas on what PI is or should be ( Table 3). Debate begins with the merits of integration versus specialisation. Peters *et al.* (2018; see also Daviter, 2017) outlines reasons to not prioritise collaboration if:

• it undermines benefits of specialization
• it limits the freedom of scientists or sources of innovation
• overlaps and ‘redundancies’ are useful, to maintain resilience
• information sharing across public agencies undermines privacy and civil liberties
• it fudges accountability.

**Table 5.** Contested requirements for policy integration.

*How to proceed*	Demonstrate why integration would be more effective than specialisation Foster networks and collaboration or hierarchy to promote integration
*Trade-offs between strategy*	Foster high-level political debates to boost the authorizing environment Maintain informal ways to seek long-term agreements and windows of opportunity
*Co-existing strategies will have:*	Negative consequences (e.g. national performance measures undermine autonomous local collaboration) Positive consequences (e.g. hierarchical direction, network collaboration, market innovation)
*Proxies of success include*	Formal evaluation: a clear link between stated intentions and outcomes Autonomy: meaningful decentralisation of authority Vision: a formal strategy with clear intent and steps to delivery Ownership: stakeholder awareness, inclusion, and buy-in Satisfaction: customer and job satisfaction with ‘one stop shops’

Then, there is contestation on the best means to integrate: maintain *networks* of specialist policy actors across and outside of government, and foster *collaboration* to establish a common framing and response to a problem; or, assert *hierarchy* via formalised means of centralised coordination, such as to set strategy, legislate, establish conditionality for funding, appoint a ‘czar’ to oversee delivery, or use internal markets for public sector competition (Peters, 2018: 6–9). There are also dilemmas about the trade-offs between high-level debates among elected politicians, to boost the authorising environment for change, and low-level means for officials to broker agreements and work for the long-term ( Deslatte *et al.*, 2017: 430; Deters, 2018: 367; Hrelja, 2015:12; Rietig, 2019: 244; Vantaggiato, 2020: 654; 665).

In practice, these approaches co-exist. The choice is not simply to pick one but also consider its impact on others (e.g. Daviter, 2017; Vanhoonacker & Neuhold, 2015). Negative consequences occur when hierarchical performance measures or internal markets create competition between actors who would otherwise collaborate, cultures develop within specialist networks that thwart centralisation, or a lack of collaboration reduces the exchange of policy relevant information and ‘ownership’ of policy ( Peters, 2018: 8; see also Jordan & Maloney, 1997). Or, there are mutual benefits. Wong (2019b: 7) describes reinforcing roles during SDG integration: networks maintain relationships between actors to cross sectoral boundaries, build capacity, and deliberate; hierarchy provides direction and resolves disputes; and a ‘market’ for ideas aids innovation (see also Nunan *et al.*, 2012).

This contestation between approaches exacerbates uncertainty. Sophisticated formal evaluations of success are rare, the results are inconclusive, and measures of coherence are unclear ( Candel, 2017; Careja, 2011; Jordan & Lenschow, 2010: 155; May *et al.*, 2006: 383; Trein *et al.*, 2023: 9–10). Studies engage with the idea of success but express high uncertainty on how to avoid unintended consequences (e.g. Rayner & Howlett, 2009; Vince, 2015). A range of proxy measures of success can include:

• Meaningful decentralisation of authority and widespread awareness and buy-in ( Simeonova & van der Valk, 2010: 1424–25; 2016).
• A clear vision and sense of shared responsibility ( Simeonova *et al.*, 2019: 126–30).
• Participant awareness and ownership ( Indig *et al.*, 2019: 23).
• Employee and citizen satisfaction with ‘one-stop shopping for government services’ ( Blackburn, 2014; Blackburn, 2016).

***Carey et al.’s facilitators of WG or JUG***

These elements – uncertainty, co-existence, trade-offs – inform pragmatic advice on how to join-up policymaking. Rather than a simple how-to guide, the emphasis is on balancing multiple concerns during continuous action. Carey and colleagues identify ‘top-down’ and ‘bottom-up’ dynamics to support a flexible:

1. *Vision.* Establish an overall purpose tailored to local contexts.
2. *Source of direction.* Highly centralised measures are inadequate. Governments rely on actors across and outside of government.
3. *Plan for action in multiple ‘operational’ levels.* Secure high-level support for ‘strategic government’, and support ‘champions’ in ‘managerial’, ‘practitioner’, and ‘community’ contexts.
4. *Approach to organisational change.* Maintain multiple functions, including to create units for ‘shared leadership’ while noting that new arrangements can undermine collaboration. Maintain relationships between managers of each organisation, adjust regulatory and budget processes to align with the vision, and work continuously on ‘cultural and institutional change’.
• *Mix of ‘hard’ and ‘soft’ measures.* Hard measures produce a ‘mandate for change’, ‘accountability and incentive mechanisms’ suitable to the task, and ‘dedicated resources’ to be used flexibly at local levels. Soft measures influence cultural practices such as to support collaboration, training, and information sharing ( Carey *et al.*, 2014: 7–9; Carey & Crammond, 2015: 1026–27; see also Eppel & Lips, 2016: 41; Vincent, 1999).

**Figure 2.**
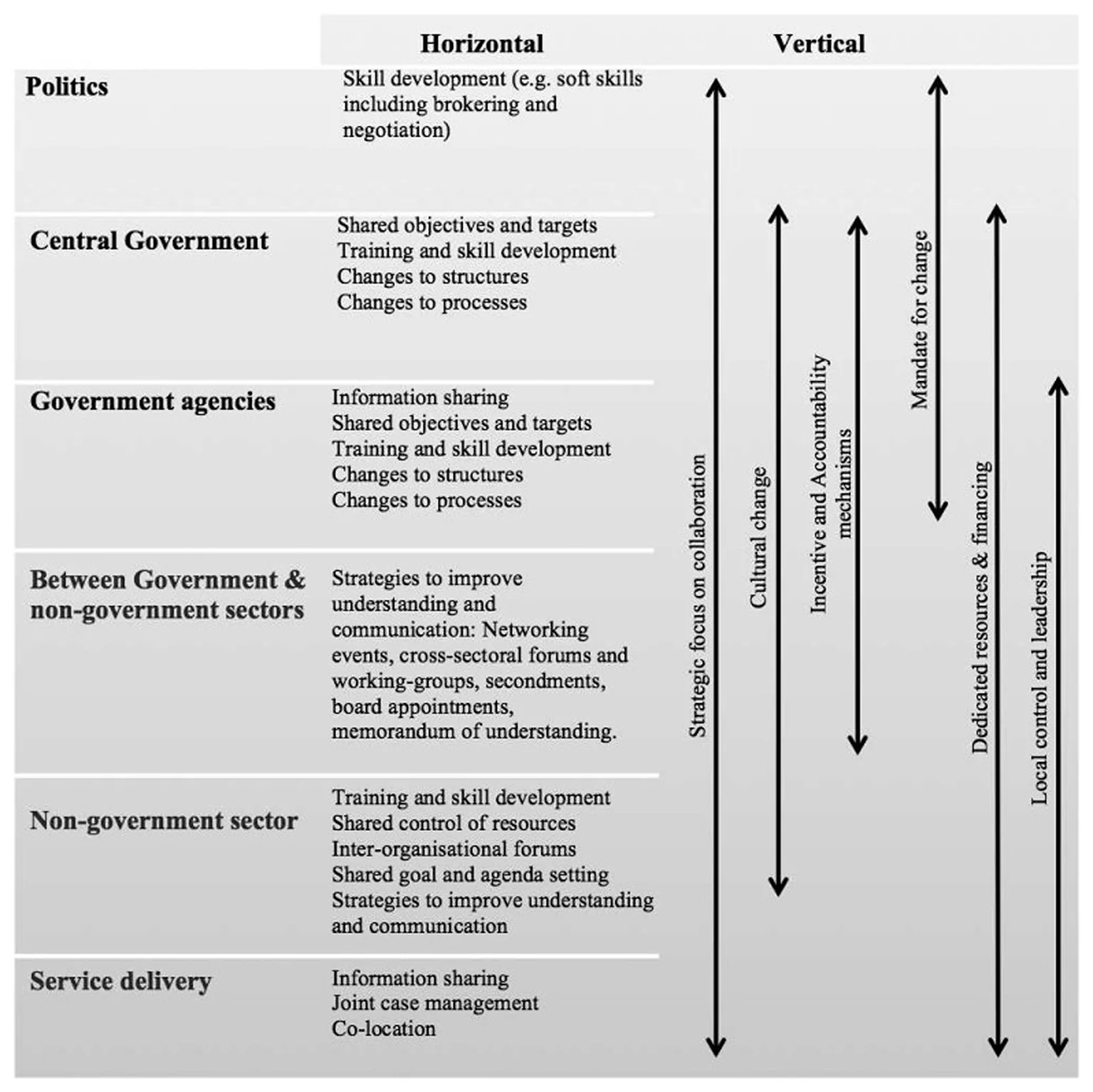
Carey’s ‘evidence-based heuristic for joined-up governance’. Source: Carey (2016: 78).

Carey (2016: 78) visualises these dynamics in Figure 2. For example, ‘soft’ skills are essential to cooperation across all contexts: central governments may focus more on structural and process changes, and others may be essential to networks, with all contributing to ‘cultural change’ and a ‘mandate’ for change.

***Facilitators of policy integration***

Table 6 identifies broadly similar facilitators as in Carey *et al.*’s work (and in our review of systems leadership - Cairney & Toomey, 2025). These facilitators relate to multiple contexts characterised by varying levels of centralisation, public body autonomy, trust and collaboration cultures.

**Table 6.** Facilitators of policy integration.

*Vision*	Establish a clear rationale and strategy for change (short- and long-term)
*Leadership in networks*	Boost the authorising environment for change Invest in collaborative capacity Foster and use relational skills
*Boundary spanning*	Adapt to varying rules, norms, and expectations in each context Help actors understand and reduce barriers to cooperation
*Supportive architecture*	‘Hard’ measures to reform and regulate ‘Soft’ measures to foster high-trust relationships and collaboration

***An overall vision***

‘Vision’ can relate to high-level aims shared across organisations, sectors, a country’s government or public sector, or multi-level jurisdictions. A long-term vision is important given PI timescales. Otherwise, actors may be dispirited by the lengthy gaps between an impetus for change, coherent strategy, and progress in implementation. Analyses of action during crisis also show that a clear vision can be produced quickly when the issue generates attention and the political rewards for action are high, prompting high expectations for joint-working and for the blocking power of vested interests to diminish ( Carayannopoulos, 2017; Carayannopoulos & McConnell, 2018: 357). If so, one strategy is to present more enduring wicked problems as extraordinary, urgent, and escalating threats that are not served by the status quo, prompting the need for rapid change and new ways of thinking (2018: 365; Blackburn, 2014, 2016; Löschner & Nordbeck, 2020: 11). Another is to relate vision to (1) high ideals, such as a public service’s ‘spirit of service to the community’, and (2) the sense that delivery is feasible, such as by identifying a few key priorities, backed by targeted and high quality analysis (as in New Zealand approaches - Scott & Bardach, 2019: 200–2; Scott & Boyd, 2023; Scott & Macaulay, 2020: 579; Washington & Mintrom, 2018: 31–2).

***Leadership in networks: fostering collaborative capacity and trust***

Our review of systems leadership ( Cairney & Toomey, 2025) emphasised the need for training to foster new attributes, mindsets, and skills essential to foster collaboration and trust between participants. The aim is to not only to secure support from the most senior people in government (e.g. Treffgarne, 2019) but also distribute leadership among many actors in a system, such as to harness the ‘energy, enthusiasm and commitment’ of many well-trained actors, including ‘champions and sponsors’ ( Vincent, 1999: 51). This is not heroic top-down leadership, but rather the skill to facilitate collective action. There is a continuous need to nurture ‘soft’ relational approaches in leaders, to bring people together, harness collective skills, and maintain a contextual or institutional memory for joint work ( Carey & Harris, 2016: 116; see also Murti *et al.*, 2020: 7). Partnerships may need senior leaders to provide the authority and resources to invest in relational approaches ( Boston & Pallot, 1997: 394; Carstensen & Bason, 2012; Scott & Thurston, 2004; Waterhouse & Lewis, 2004: 371). This investment would connect ‘system capacity’, including shared ownership of aims backed by legislation and data, with ‘human capacity’, including multiple leadership and ‘soft skill’ roles (political, administrative, mediation, boundary spanning, change agents) ( Von Heimburg & Hakkebo, 2017: 75).

Indeed, some articles describe success in relation to systems leadership-style approaches. O’Flynn *et al.* (2011: 250–53) cite Bardach (1998) on a ‘craftsmanship approach’ including: listening, accommodating and including others, giving colleagues the power to innovate and take risks, and maintaining a clear link to local communities via staff recruitment and network management (see also Ross *et al.*, 2011: 137; Schmied *et al.*, 2010: 3522). Delany-Crowe *et al.* (2019a: 180–3) describe high trust in a ‘process’ to foster collaborative HiAP approaches in South Australia, such as to build on fruitful cooperation and reciprocity between individuals or organisations, ensure that the strategy reflects shared goals, and assure participants that they can communicate directly with senior policymakers. Further studies emphasise success in ‘capacity-building’ ( Connolly & Pyper, 2020), evidence-informed collaboration ( Samaratunge *et al.*, 2017), and relational skills of policy ‘champions’ Wamsler *et al.* (2020: 3–8).

Others highlight the role of hierarchical or elected political authority to facilitate concerted action or overcome barriers. For example, a successful WG approach to immigration in Portugal required (1) clear leadership and collaboration based on an enduring cross-political-party consensus that extends to positive relations between ‘politicians, bureaucrats, and civil society groups’, then (2) the use of this consensus to give organisations the ‘autonomy and political support’ to collaborate and innovate without risking political fallout ( Cook, 2018: 771; see also Askim *et al.*, 2009: 1022; Christensen *et al.*, 2014: 448; Lægreid & Rykkja, 2015: 974 on appointing a senior leader to mitigate contestation arising from WG reforms).

***Boundary spanners***

Boundaries ‘can be social, cultural, or professional, and relate to forms of knowledge production, geography, or levels or types of government’ ( Cairney & Toomey, 2025: 15, citing O’Flynn, 2013: 13). The literature describes attempts to cross *geographical borders* ( Janßen *et al.*, 2018) or *policy sectors* ( Kim & Bramwell, 2019) or brokerage to connect climate mitigation and adaptation actors ( Locatelli *et al.*, 2020: 367–8). Boundary problems can be:

1. *Practical*, including the lack of resources to gather data or work together well (e.g. Paylor & Simmill-Binning, 2004: 337; Signoretta & Craglia, 2002: 71–2).
2. *Political*, including a lack of trust which contributes to excessive secrecy, poor communication practices, and contested notions of joint aims ( Ling, 2002: 619–21; e.g. Hjalmarsson, 2015: 187; Humalisto, 2015: 156; Pfau *et al.*, 2017 on ‘biofuels’).

Carey *et al.* (2017) describe ‘boundary spanners’, connecting different ‘cultural’ and ‘functional’ dynamics within or across government departments or units. These facilitative and translation roles are essential even following formal reforms, which do not remove the need for informal collaboration or the potential for turf wars (see also Crosbie *et al.*, 2018: 10; Hoggett, 2003: 120; Larner & Craig, 2005; Lips *et al.*, 2011). Jackson and Stainsby’s (2000: 13–15) account of *network governance* signals similar skills and mindsets, including: diplomacy and conflict resolution, brokerage, and facilitation. Smucker *et al.* (2020: 12) also describe *bridging organisations*, using methods for collaboration (‘participatory vulnerability and capacity assessment’) to boost local networks of public and private actors. Conversely, a tendency for public health advocates to use their own language of ‘evidence-based argument’ to justify action, rather than making a normative case to their wider audience, reduces traction across government ( Crammond & Carey, 2017; Nutbeam & Boxall, 2008: 753; Nykiforuk *et al.*, 2019).

***A supportive architecture and environment for new strategies***

A supportive ‘architecture’ changes ‘incentives, provides authority, builds long-term trusting-based relationships, and recognizes and rewards cooperative behaviors’ ( O’Flynn *et al.*, 2011: 248). It consists of ‘hard’ or ‘structural’ measures to provide ‘political backing’ for reform and ‘soft’ measures to foster collaboration ( Candel, 2017: 531; Carey *et al.*, 2014: 9). This architecture is apparent at different scales of activity, including:

• To share responsibilities across countries (e.g. Tolley *et al.*, 2016: 6–10 on a ‘dashboard’ to monitor progress).
• To coordinate central government departmental responses (e.g. Babel *et al.*, 2015: 860 on intensive coordination on migration).
• To empower subnational or local action (e.g. Aurich-Beerheide *et al.*, 2015: 390; Rauken *et al.*, 2015: 419; Sullivan, 2003: 366; Willems *et al.*, 2021: 84 on the need for local resourcing and autonomy to combine coherent delivery with direct representation and citizen participation).
• To share responsibility within an organisation (e.g. Andersson & Liff, 2012: 848–9 on ‘multiprofessional teams’).

Here, the metaphor ‘architecture’ can have subtly different meanings. In some cases, ‘architecture’ suggests that a supportive process can be designed anew. For example, a South Australia model often provides positive lessons when demonstrating the value of leadership and accountability, clarity, assigning resources, supportive networks, and good communication ( Ballintyne & Mintrom, 2018; Baum *et al.*, 2010; Cairney *et al.*, 2021a; van Eyk *et al.*, 2020: 968). In other cases, ‘architecture’ suggests something more established on which to build further. For example, Christensen *et al.* (2007: 391–2) relate WG reforms to a long-term Norwegian context characterised by supportive factors, including consensus seeking political parties, a ‘significant tradition of local self-government’, civil service leeway resulting from ‘high levels of mutual trust and shared attitudes and norms among political and administrative leaders’. New WG measures interact with established cultures in more-or-less autonomous levels or types of government (2007: 394–5). We can situate other cases within that range of old and new cultures. For example, Luan’s (2010) account of successful water policy in Singapore cites long-term plans facilitating shorter-term decisions. Plowden’s (2020: 160) explanation of the success of London’s Healthy Streets programme also signals a legacy on which to draw.

### What are the barriers to policy integration?

**Figure 3.**
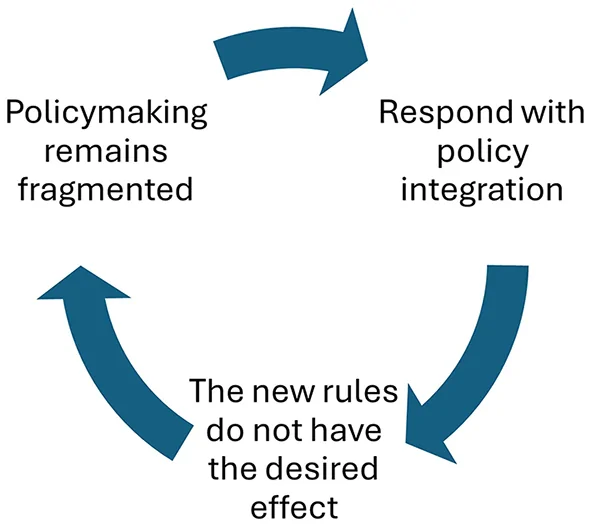
The policy integration cycle.

I introduce Figure 3 to describe a cycle in which the problems prompting reform also undermine it: complex policymaking systems seem to drive fragmentation, a new PI initiative does not have the desired effect, and the rediscovery of fragmentation produces a new impetus for integration (compare with Serrao-Neumann *et al.*, 2014: 491). Further, the production of multiple new and unconnected PI strategies may *exacerbate* this fragmentation in central government departments ( 6 & Peck, 2004) or public service delivery ( Davies, 2009: 86).

Table 7 summarises many variations on this theme, such as to describe low ‘political will’ to overcome this dispiriting cycle; a sense that integration seems more possible during formulation than implementation; and, a focus on policy subsystems which exhibit varying commitments to each other’s integration agenda ( Cejudo & Trein, 2023: 12).

**Table 7.** Barriers to policy integration.

*Evergreen drivers against integration*	High-level efforts are designed to not threaten the status quo More substantive efforts provoke successful opposition Entrepreneurs and champions can make a modest difference Integration efforts expose competing goals, rules, incentives
*New barriers following NPM*	Hierarchies, silos, internal competition, service delivery fragmentation
*Merging new policy with the old*	New rules are layered onto existing institutions New instruments are added to an existing mix
*PI measures lack clarity, congruity, and capacity*	Reform agendas are vague (by accident or design) New aims do not fit with routine government business There are competing ‘logics’ to policymaking There are limited resources to collaborate or coordinate (an amplified implementation gap) Strategic aims are clear in theory but unclear or contested in practice
*Unclear accountability and measures of success*	PI fudges policymaking responsibilities and accountability The overall effect of multiple forms of accountability is unclear Political measures of success may trump programmatic aims
*Top-down approaches are inadequate*	Many governments persist with a top-down view of PI Ill-resourced top-down initiatives have a limited local impact Centralised approaches do not boost local capacity and collaboration
*Sectoral dominance: some aims are more important than others*	Win-win strategies have limited success Economic growth remains the main priority Environmental and public health aims need to support that priority International peace and development has symbolic support National security and stability has substantive support

***There are evergreen drivers against integration***

Policy sectors have their own agendas, subsystems produce their own policy instruments, and public or non-governmental organisations implement and evaluate policies with reference to ‘their own priorities and targets’ ( Cejudo & Trein, 2023: 12). This dynamic suggests that: ‘any policy integration initiative may fall victim to the dominance of subsystem logic of policymaking, as actors are likely to dismantle, dilute, or neutralize efforts toward integrated policies during agenda-setting, decision-making, and implementation’ (2023: 12). Cejudo and Trein (2023: 15–21) relate this context to a pattern of activity characterised by:

**1. High-level strategic action**

Heightened concerns about ‘wicked’ problems are often addressed at high levels of political systems, or grand-scale international agendas.

**2. A vague response, producing limited opposition (and capacity)**

Such processes produce vague strategies that provoke limited opposition or conflict among actors with competing interests, but also provide limited new capacity. Coordination strategies avoid conflict and emphasise information sharing and learning ( Rietig & Perkins, 2018: 502; Russel *et al.*, 2020: 15; Wong & van der Heijden, 2019: 841–3). Policy instrument strategies rely more on ‘soft’ than ‘hard’ measures ( Pollack & Hafner-Burton, 2010: 292). Softening strategies include ‘watering down, issue redefinition, and the setting of targets without actions’ ( Deters, 2018: 359; Sotirov *et al.*, 2020: 19). The result is a window of opportunity for a general strategy with limited follow-through and the postponement of debate on competing objectives.

**3. Occasional attempts to change the status quo provoke opposition**

Some may seek to reallocate capacity and challenge the usual ways of working, provoking more opposition in established subsystems. If so, actors in subsystems can constrain progress, such as if the number of ‘veto players’ increases whenever joint action is necessary (and policymakers in each subsystem prioritise their ‘clients’ over integration - Howlett and Rayner, 2021).

**4. There is *some* scope for key actors to make a difference**

Some actors may facilitate progress, such as the entrepreneurs or brokers, in formal or informal roles, seeking opportunities for policy change, collaboration, or to offer integration with their sector as a solution to another sector’s problem ( Bocquillon, 2018; Garcia Hernandez & Bolwig, 2021: 74; Kim, 2014: 334; Maltby, 2013; Namugumya *et al.*, 2020: 5; Rietig & Perkins, 2018; Storch & Winkel, 2013; Svensson, 2019; Vantaggiato, 2020). Local ‘public managers’ and ‘street-level bureaucrats’ may also be influential if given the flexibility and autonomy to collaborate with stakeholders ( Cejudo & Trein, 2023: 21; Lambelet, 2023: 163; Sevä & Sandström, 2017).

**5. Competing goals and incentives remain**

In this fragmented and competitive context, policy actors respond to contradictory incentives. Many welcome WG in principle but in practice assign most ‘policy capacity’ to more pressing departmental or sectoral incentives, rewards, and accountability mechanisms ( Flinders, 2002: 56–7; Kavanagh & Richards, 2001: 9; Hughes *et al.*, 2015: 233). The will to ‘share services’ may be strong but comes with risks associated with separate budgets and core objectives, different rules or cultures that are difficult to align, and limited knowledge of how to manage when there is little training or experience on which to draw ( Valkama *et al.*, 2016: 32–4). This dynamic is apparent in each level of government, including:

• *International level: delivering the SDGs.* Strategies have long lists of aims, without a proper sense of priorities or trade-offs ( Azizi *et al.*, 2019: 446–7). Lead units formulate strategy without sufficient collaboration with others, which limits clarity on jurisdictional responsibilities ( Happaerts, 2012; Pilato *et al.*, 2018) and delays inevitable ‘turf wars’ between actors representing environmental, economic, and social concerns. Such problems emerge during implementation ( Abazaj *et al.*, 2016; Babu *et al.*, 2018).
• *Supranational level: examples from the EU.* Some countries treat EU Directives as top-down measures requiring expert-informed compliance; others as opportunities to foster meaningful stakeholder involvement ( Atkinson & Klausen, 2011: 239; Elbakidze *et al.*, 2015). New hard instruments are layered onto old systems ( Indset & Stokke, 2015: 980; 995). The idea of integrating economic and environmental aims has strong symbolic or rhetorical value, but shows minimal progress when economic drivers dominate ( Winkel & Sotirov, 2016). The rhetoric for integration aids ‘disintegration’ by providing a useful veneer to cover inactivity (2016: 510). Long-term progress relies on partnerships between specialist EU and member state agencies that are vulnerable to national horizontal integration ( Egeberg & Trondal, 2016).
• *National and subnational dynamics.* Cairney *et al.* (2021b) describe multi-level GM in which responsibility is distributed across a system and shared by different levels of government (2021: 419–21; see also Shill *et al.*, 2012: 168). Policy incoherence is baked into policymaking design, since shifting responsibilities up to the EU and down to Scotland allowed each level to go its own way with only some obligations to cooperate and cohere. Partisan competition in national and subnational contexts creates tensions, such as when the Scottish Government describes being stymied by relative UK inaction.

Overall, such insights signal an uncertain connection between the short-term impetus for integration and the longer-term dynamics when normal service resumes.

***There are new barriers following NPM***

WG often represents a top-down response to the unintended consequences of NPM reforms, but the NPM legacy remains. Managerial hierarchies, silos, internal competition, the delegation of administration to agencies, and the shift of responsibility for policy delivery to a mix of public, private, and third sector organisations, still undermine collaboration and coordination across government and the public sector. Governments face the choice between soft measures of coordination which will gain little traction in competition with the core business of government departments, or another round of painful structural reforms with uncertain effects ( Christensen & Lægreid, 2007: 1063). The consequence is that many governments have used the same vague WG language but overseen different approaches with unclear short-term results (2007: 1063–4; e.g. Askim *et al.*, 2009: 1022; Aulich & Hein, 2005: 43; Christensen *et al.*, 2014: 447).

Laffin *et al.* (2014: 763–5) also identify low expectations for relatively decentralised or pluralist network governance forms of WG. This hope ignores ‘the realities of contemporary public service delivery’ when policy remains driven by hierarchical government and economic or business interests are more powerful than others (e.g. Lance *et al.*, 2009: 253, citing Ling, 2002: 626; Whitehead, 2003: 12; 6, 2004: 126).

***Merging new policy with the old: there is no blank page***

Rayner and Howlett (2009: 99–100) relate policy design to ‘the legacies of past policies’ that ‘constrain present opportunities’. Designing a policy mix is ‘about re-aligning or de-aligning and replacing certain elements of established regimes and overcoming the “stickiness” of these elements is critical to the success of policy integration’. Studies of new institutionalism show how policymaking rules change through modification rather than simple replacement, when:

• new rules are added to the old (‘layering’)
• old rules no longer address a changing problem or new goals (‘drift’)
• different rules or instruments are repurposed to address established goals (‘conversion’)
• a different (established) institution is used to solve the problem (‘displacement’)
• or, a policy regime collapses when it proves to be un- or counter-productive (‘exhaustion’) (e.g. Thelen, 2004 in Rayner & Howlett, 2009: 103).

The result could be incoherent policy design if layered slowly onto existing policies rather than redesigned with urgency and new capacity ( Vince, 2015: 162; 174–5). Or, there could be transformative change, but with no guarantee that it relates to coherent intent. Rayner and Howlett (2009: 99–100) show that budding integrators face an ‘insidious’ problem when a policy mix has survived through ad hoc modifications, produced by different actors for different political purposes, and contributing to their complexity, costs, and resistance to change ‘since even dysfunctional elements of existing regimes can confer benefits on well-entrenched interests who may resist their alteration or elimination’ (2009: 100; 103). Or, reformers can exploit a clearer but rarer opportunity for ‘replacement’ following ‘exhaustion’ and a widespread perception of regime failure (2009: 103). In other words, modify a modestly successful approach or wait for a window for redesign following major failure. Of course, determining the success of current policy, and how to respond, is an exercise of power, not a technical act of evaluation (e.g. Sotirov & Storch, 2018: 986–7).

These issues are magnified when a policymaking system contains many cross-cutting plans that are not well integrated, contributing to coordination fatigue ( Isaksson *et al.*, 2017; Pengilley & Kelly, 2018: 3). Or, a push for coherent spatial planning, such as to promote the equitable allocation of public services, may be low priority or a vote loser ( Santinha, 2016: 173; Schmitt & Smas, 2023 and Storbjörk & Hjerpe, 2014).

***PI agendas and strategies lack clarity, congruity with routine government business, and the capacity to deliver ambitious aims***

Our reviews draw on Cairney *et al.* (2022b) to describe three limitations to progress: low clarity, congruity, and capacity. A policy means too many things to too many people, is difficult to reconcile with routine government business, and lacks the political and systemic capacity to ensure proper delivery or implementation. Competing interpretations argue that:

**1. Low clarity, congruity, and capacity can be avoided or overcome.**

Studies focus on the weak leaders who make vague promises without understanding the evidence, changing the rules to accommodate policy change, or assigning resources. Such studies bemoan:

• *Symbolic policy or coordination.* Policymakers use the WG language with no intention of further action (e.g. Desrosiers & Lagassé, 2009; Dyrhauge, 2014: 985; Ekhaugen, 2022).
• *Insufficient political will to follow-through.* When policymakers face barriers to progress, they do not have or use ‘political will’ to overcome them (e.g. Agrawal *et al.*, 2021; Briassoulis, 2011; Cavallo *et al.*, 2017, 2018; Colesky & Raath, 2015; Downs *et al.*, 2015; Dupont & Primova, 2011; Farmery *et al.*, 2020; Negev, 2016: 217; Scobie, 2016: 27; Yin *et al.*, 2016: 84; Zhou & Seethal, 2011: 401). Some describe corruption or hypocrisy and low citizen trust in policymakers (e.g. Danaeefard *et al.*, 2019: 70–72).

**2. Low clarity, congruity, and capacity are evergreen features to which to adapt.**

Studies describe these limits as features of policymaking systems: uncertainty is inevitable and assigning meaning is an exercise of power; there are too many rules to accommodate; and, capacity is largely about reassigning tasks and collaborating across organisations (see also Biesbroek *et al.*, 2020; Collste *et al.*, 2017; Guerrero & Castañeda, 2021 on technologies to reduce uncertainty). Then, they emphasise the politics of coordination to avoid a technocratic language of collaboration and ‘evidence based’ policy designed to somehow overcome politics and ideology ( Davies, 2009: 83). Problems may include:

• *Superficial agreement.* Organisations use a vague vision, but ‘shallow consensus’ is not enough to contain or overcome ‘political value conflicts’ ( Davies, 2009: 80). Conflicts may be party political, or subtler differences between rules used by public bodies to act and evaluate progress (2009: 87).
• *Mainstreaming overload*, when multiple strategies or elements of each strategy compete for attention ( Nunan *et al.*, 2012: 265, citing Agrawala & Van Aalst, 2008).
• *Cultural differences.* Less visible tensions arise between actors committed to change, but facing routine barriers to cooperation associated with sectoral organisation and budgets, perspectives and conceptual models, and measures of progress ( Waylen *et al.*, 2019: 11; White *et al.*, 2020: 500).

**A lack of clarity: the double-ambiguity of PI**

PI may exhibit *double-ambiguity* when policy aims are unclear and a WG approach is vague ( Ross *et al.*, 2011: 135). This lack of clarity kicks the can down the road, and may exacerbate ‘constant power struggles between different authorities’ during implementation ( Söderberg, 2016: 90). Or, ambiguity may be used strategically to limit follow-through ( Candel, 2017: 530; Casado-Asensio & Steurer, 2016a), allow different elements of policy to appeal to competing actors (e.g. Borrass *et al.*, 2017: 22), and avoid full scrutiny ( Walker, 2001, 2009; Warren, 2012; Wines & Scarborough, 2015: 136; Wise, 2006: 70). Either way, the inability to foster clear WG approaches, and assign ‘principled priority’ to the aim, contributes to a lack of coordination and commitment ( Aall *et al.*, 2015: 979; Kissinger *et al.*, 2021: 91; citing Lafferty & Hovden, 2003).

In some cases, research focuses on the need to reduce ambiguity by addressing a *knowledge-deficit among some actors.* The solution is to explain their preferred frames better and improve the use of scientific models when they are too technical for most policymakers to understand (e.g de Smedt, 2010). HiAP research characterises this approach, to describe low ‘public health literacy’ and a lack of knowledge of social determinants of health ( Carmichael *et al.*, 2012: 189; Pengilley & Kelly, 2018: 4). This knowledge-deficit contributes to a lack of integration in health and equity strategies when a *social determinants* frame is not consistent with an *individual lifestyles* frame ( Delany-Crowe *et al.*, 2019b: 10; Pescud *et al.*, 2019: 8; Peters *et al.*, 2016: 294). The problem is further exacerbated by the ambiguity of ‘integrated public health policy’ ( Tubbing *et al.*, 2015), and absence of ‘clear operational criteria and defining characteristics’ ( Hendriks *et al.*, 2014: 174).

In other cases, the dynamic reflects *underexplored ambiguity.* Hezri and Dovers (2009: 303) find a lack of progress on ‘ecological sustainable development’ because it ‘is ambiguous and fraught with contradictions’. Similarly, the food-water-energy ‘nexus’ has broad rhetorical support but is difficult to translate into practice, especially if it does not address tensions between separate sectoral ‘mandates’, ‘expectations and objectives’ or assign clear roles for integration ( Venghaus & Hake, 2018: 190). See also interpretations of ‘landscape’ in policy and planning ( Keller & Backhaus, 2020: 113; Kristensen & Primdahl, 2020; Sandström & Hedfors, 2018; Svensson *et al.*, 2020; van Oosten *et al.*, 2018).

Or, ambiguity reflects *visible contestation* when actors define aims for their purposes (e.g. Tosun & Leininger, 2017 on the SDGs). Jordan and Lenschow’s (2010: 148–53) analysis of EPI progress finds major disagreements on what it represents, such as a broad ambition or detailed strategy, and a means to coerce or encourage change. Contested meaning contributes to varying commitment and approaches across time or countries, and a lack of embeddedness ‘into everyday political practices’ (see also Biesbroek *et al.*, 2010; Kettner *et al.*, 2015; Kettner & Kletzan-Slamanig, 2020). Aubrechtová *et al.* (2020: 13) describes different sectors pursuing different conceptions of a problem (e.g. ‘biocentric’ or ‘anthropocentric’). A lack of clarity can also reflect disagreement and compromises that suit no actor fully (e.g. Lajeunesse, 2021). Or, vague terms like complexity and WG hide political choices, such as to exclude some experts and take some issues off the agenda ( Ulijaszek, 2015: 224).

These perceptions of the causes of ambiguity inform competing views on how to resolve it. One response is to seek agreement on a more attractive term that actors can get behind. However, the unintended consequence is a hodgepodge of faddish and unconnected terms ( Cairney *et al.*, 2021a on HiAP; Bahn-Walkowiak & Wilts, 2017). Entrepreneurs can also use ambiguity to rally initial change but struggle to institutionalise substantive responses (e.g. Bocquillon, 2018). In that context, studies seek centralised or decentralised means to boost clarity, including:

• *One authority should clarify what to do, and how, including standards, roles and responsibilities.* Avoid ‘vague and general’ strategic priorities that are not the responsibility of any named organisation, and serviced by low-capacity internal coordinating units, if it allows more powerful departments to ‘fudge’ their accounts of progress ( Boston & Pallot, 1997: 396; Yusuf *et al.*, 2018). Address a lack of clarity regarding the role of a leader or ‘focal point person’ to join-up policy and services ( Cleaver *et al.*, 2020: 5; Falaleeva *et al.*, 2011; Homel, 2005: 365; Mahlangu *et al.*, 2018: 309; Reeve *et al.*, 2018; Williams, 2019; Yusuf *et al.*, 2022: 495).
• *Reduce ambiguity via wider engagement and collaboration.* Foster discussion in national and local government to agree on shared aims ( Bigdeli *et al.*, 2020: 3–4; Caudle & Spiegeleire, 2010: 20; Davies, 2009: 89; Naidoo, 2013: 399).

Some accounts suggest that this ambiguity can be reduced over time, such as by exploiting crisis and windows of opportunity for change. MacCarthaigh and Boyle (2011: 219–20) describe the Irish government’s initially loose and un-strategic use of the WG language, which reflected a tendency to ‘oscillate between preferences for specialization and integration’. By 2015, it had overseen ‘unprecedented and systemic public sector reforms’, partly on the back of economic crisis ( MacCarthaigh & Hardiman, 2020: 179).

**A lack of congruity: new expectations interact with how things work around here**

Reformers have a notional choice between maintaining vague aims to bolster a superficial consensus or clear and substantive change that will provoke opposition. Many examples find visible ‘turf wars’ ( Baum *et al.*, 2010: 479; Bizikova *et al.*, 2015; Karré *et al.*, 2013: 71; Rosenberg, 2020). Some find less visible cultural differences or routine processes of maintaining business-as-usual ( Boothby, 2017: 35; Cashmore & Axelsson, 2013: 10; Essex & Carmichael, 2017: 274).

In some areas, we find a wider tension between competing policymaking ‘logics’. For example, centralised and top-down may compete with decentralised and polycentric strategies on the same issue ( Sandström *et al.*’s, 2020). Or, in environmental policy there may be a disconnect between the choice to favour boundaries based on territorial autonomy and ‘administrative logic’ versus an ‘ecological logic’ when nature does not fit into human-designed boundaries (2020: 7–8; Satumanatpan & Chuenpagdee, 2015: 13; Stjernström *et al.*, 2018). Further, Russel *et al.* (2018b: 1360–61) identify multiple competing ‘logics’ of appraisal associated with different levels (e.g. local, national, supranational) or types of governing organisation, across policy sectors and professions, and in theory versus practice. One dominant political logic may encourage the *performance* of appraisal, often by consultants depending on this work, for symbolic purposes rather than to reflect on programmatic success (2018: 1364–65).

**A lack of capacity: integration gaps akin to implementation gaps**

This limitation may relate generally to a gap between aspirations versus resources for collaborative policymaking ( Bishop & Flynn, 1999: 74; Caudle & de Spiegeleire, 2010: 9; 17; Young & Grant, 2015), a failure to reallocate resources when reorganising responsibilities ( Sheperdson *et al.*, 2014), or unanticipated problems such as the poor use of ICT to facilitate WG (see Cairney, 2025b). This limitation may also follow legislation, such as when multiple countries passed Climate Change Acts to strengthen international commitments, but lacked sectoral targets or meaningful sanctions for slow progress ( Nash & Steurer, 2019: 1061–62; see also De Roeck *et al.*, 2018: 40; Zingraff-Hamed *et al.*, 2020: 466–70). The effect of limited capacity and coordination may be cumulative and demoralising (6 & Peck, 2004: 9; see also Blockley, 2010: 198; Signoretta & Craglia, 2002: 71–2).

This focus on capacity resembles a traditional focus on implementation. The rather limited study of decentralised or bottom-up capacity does not hold out much hope for progress (e.g. Casado-Asensio & Steurer, 2016b: 275; Hoff *et al.*, 2019; Persson, 2013; Steurer & Clar, 2015; Walker *et al.*, 2015). However, most research adopts a relatively top-down perspective in which PI requires the initial integration of goals, and coherence of instruments, then consistency of application ( Lambelet, 2023). If so, there is always a gap between PI strategy and delivery (e.g. Chaya *et al.*, 2019; Wretling *et al.*, 2018), such as in the absence of a senior leadership with a clear ‘mandate’ to overcome ‘turf wars’ and other problems ( Glasson *et al.*, 1997; Kavanagh & Richards, 2001: 10–12; Karré *et al.*, 2013: 71; Kendig *et al.*, 2014; Wilson & Wileman, 2005).

Political dynamics change when shifting from PI design at a supranational or national level to delivery at a local level or within each policy sector, enabling different ways to frame the policy problem, set goal priorities, and manage interests in networks, or prompting blame game strategies to avoid responsibility for the failure of a strategy designed elsewhere ( Candel, 2017: 530; Jordan & Lenschow, 2010: 153; Neto *et al.*, 2018; Nilsson *et al.*, 2012; Sarti, 2023; Widmer, 2018: 77). This capacity gap may also reflect insufficient engagement with key actors across government ( Kayiwa *et al.*, 2019: 176–9), and a ‘lack of inter-agency and inter-ministerial approaches for building partnerships with other stakeholders’ ( Antwi-Agyei *et al.*, 2017: 1; Antwi-Agyei *et al.*, 2018). Such obstacles should be seen as continuous features of policymaking rather than closable gaps (e.g. Biesbroek & Candel, 2020: 79; Carbone & Keijzer, 2016: 39–40; Feiock *et al.*, 2017; Namugumya *et al.*, 2020).

***Unclear accountability and competing measures of success***

Successful PI requires shared responsibility and effective measures of success. However, WG approaches complicate who is responsible for what. Christensen *et al.* (2014: 444; see also Bovens, 1998; Christensen & Lægreid, 2011; Sullivan, 2003: 355) describe competing accountability, including:

• *Political*, such as when parliaments scrutinise ministers (and connect this activity to electoral accountability).
• *Administrative*, such as when ministers use measures to direct civil servants.
• *Legal*, to regulate behaviour and ensure ‘equal treatment’.
• *Professional*, when professional bodies regulate their own members.
• *Social*, in partnerships with stakeholders or when reporting to citizens on services. In many such cases, policy actors may be key to collaboration but have no formal powers or means to be held to account ( Wilkins, 2002: 118). Or, ‘community governance’ and accountability may be a hard sell to citizens and stakeholders ( Matthews, 2014: 454).

In that context, WG measures to create new forms of accountability contribute to a fudge of measures ( Christensen *et al.* 2014: 453). ‘Horizontal’ coordination, involving many organisations collaborating, clash with ‘vertical’ measures for accountability, involving a national government setting targets or regulating delivery organisations. Shared public service performance measures co-exist with separate forms of professional accountability (2014: 445–6; 453). One-stop-shops fudge political accountability while emphasising service accountability (2014: 445–6; 450–1). New informal ways of collaborating operated alongside old ways of monitoring sectoral organisations ( Lægreid & Rykkja, 2022: 699).

Such experiences suggest that reforms are inadequate unless they address immediate administrative obstacles *and* longer term cultural changes ( Ryan & Walsh, 2004: 629). Formal reorganisations change mechanisms of accountability, and meaningful change also requires policy actor buy-in, collaboration, and external acknowledgement of the value of new forms of accountability ( Lægreid & Rykkja, 2022: 687). However, the incentives to produce such reforms are unclear whenever there are competing measures of success. Declared success can relate to a strategy’s impact on government popularity, its stakeholder legitimacy, or socioeconomic impact ( McConnell, 2010). This dynamic combines with fudged accountability to amplify the potential for failure, when actors use a WG approach ‘for political gain or blame avoidance’ rather than invest their reputations on longer-term success ( Stack *et al.*, 2018: 126–7; Vince, 2015: 162).

***Top-down or centralised approaches are inappropriate or inadequate***

Central governments do not respond well to these evergreen and inevitable constraints to PI. Their NPM reforms have created or exacerbated the problem, they have responded with unclear and ill-resourced strategies, and they tend to see the policymaking problem from the top-down, prioritising central control over local autonomy and wider collaboration.

Carey *et al.* (2015a, 2015b: 167) describe such top-down WG approaches as inadequate, since producing an integrated central government strategy is no guarantee of concerted action and coherent outcomes in local government and ‘street-level’ contexts. Local ‘implementing organisations’ become dispirited with waves of top-down reforms, and their commitment to the next initiative wanes ( Molenveld *et al.*, 2020: 9). This sense of commitment matters whenever organisations have some autonomy and discretion to use limited resources to fulfil obligations, albeit within a framework of regulation and performance management ( Molenveld *et al.*, 2021: 127–9). Their commitment to collaboration rises if included by central government in policy design and they can tailor national policy to local contexts, if they have enough autonomy and resources to innovate (2020: 9; 2021: 145) or if street level organisations exercise discretion to join-up fragmented aims ( Fisher, 2013; Oldeide *et al.*, 2021: 382). Yet, many governments persist with simplistic centralised policymaking, producing unintended consequences (e.g. Maghsoodi Tilaki & Hedayati, 2015).

### Country studies of barriers to top-down or centralised PI: UK, Australian, and Nordic experiences

The PI and WG literatures provide a wealth of country case studies on the limitations of top-down approaches. Here, I use the UK as an exemplar of a top-down approach with multiple unintended consequences, followed by Australia, then Nordic examples in which there may be greater bottom-up expectations.

*Barriers to top-down PI in the UK*

The UK exemplifies top-down approaches to PI, often studied through the lens of former Prime Minister Blair’s pursuit of joined-up government (JUG). Some accounts note that the UK was world-leading in key respects, such as to invest in research and foresight activities to anticipate complex problems ( Ulijaszek, 2015). However, most describe ill-considered and poorly resourced centralisation efforts that were doomed to fail. There are five general reasons to expect a lack of JUG progress under the Blair-led government from 1997 then Brown-led from 2007–10 ( Campbell, 2007; see also 6, 1997; 6 *et al.*, 2002; Matthews, 2013: 55–79).

First, JUG relates to the ‘Holy Grail’ of addressing ‘wicked issues’ without enough research knowledge or political agreement on their causes or solutions ( Lewis, 2011b).

Second, the UK approach was to devote limited resources to coordination. It sought to address tensions between government departments, but to ‘renovate’ existing central government structures rather than reform functions or provide new, clear and systematic incentives for departments to collaborate ( Campbell, 2007: 391–4). Rather than encouraging collaboration between well-resourced organisations in central government and the public sector, a small core executive took on key tasks and overloaded its capacity to oversee progress (before the Treasury took over – James, 2004).

Third, UK governments had concerns about blurred lines of accountability compared to the established Westminster focus on individual ministers, coupled with a lack of sophisticated performance measures for collaborative work ( Flinders, 2002: 62; Kavanagh & Richards, 2001: 14).

Fourth, the UK central government maintained contradictory objectives, to empower local public bodies and foster network governance but maintain central control ( Sullivan, 2005: 10). It was asking local public bodies in England to collaborate, but obliging health, local, and other authorities to meet sectoral targets ( Hoggett, 2003: 118; Ison *et al.*, 2018: 1215; although see Moore & Keen, 2007 and Moore *et al.*, 2007).

Fifth, the UK government’s remit is often England-only, with the expectation (from 1999) that devolved governments in Scotland, Wales, and Northern Ireland would go their own way. Further, similar PI issues arose even in contexts where a more joined-up approach was expected. In particular, there were some hopes in Scotland and Wales that devolution would bolster JUG, partly to reflect a pre-devolution legacy: ‘territorial departments had always been pioneers of non-departmentalism because of their small scale and territorial, rather than functional, focus’ ( Parry, 2012: 254). Devolution in Wales in 1999, coupled with a legal duty to ‘promote sustainable development’, and a scale and approach conducive to policy community development, prompted some hopes for a new ‘powerful force for collaborative government’ ( Bishop & Flynn, 1999: 62). However, stakeholders identified a tendency to *perform* participatory policymaking in a system where Welsh and UK responsibilities were unclear ( Chaney, 2016). Further, the Welsh government pursued multiple competing objectives, to foster collaboration and learning via local government autonomy and network governance but also exert central control and planning ( Sullivan & Williams, 2009: 166–77). Similarly, fuelled by its own story of better and longer-term partnership working and community engagement, the Scottish Government appeared to take a less centralist or hierarchical approach than the UK government to ‘community planning partnerships’ and local service delivery ( Matthews, 2014). This story widened a gap between aspirational rhetoric and reality (2014: 455–9; 468; Cairney & St. Denny, 2020). In contrast, Knox (2003) describes the initial absence of interest in JUG in Northern Ireland. The same optimism that devolved governance could improve on UK government approaches did not arise until a decade later ( Knox, 2015: 29).

Overall, the UK’s energetic approach to top-down JUG has produced a range of experiences with wider lessons. First, *a WG approach is no substitute for clear priorities or choices between competing aims.* Multiple examples highlight a superficial commitment to many aims, but with most losing out to a higher – economic or healthcare – priority ( Baggott, 2010: 147; Exworthy & Powell, 2004: 272–4; Gagnon & Labonté, 2013: 4–8; 12; Hayden & Benington, 2000: 30).

Second, *assign clear priority to your preferred modes of WG.* JUG across central and local government has a ‘superficially attractive logic’ but ‘disguises the degree to which different forms of joining up may conflict’ ( Cowell & Martin, 2003: 159). Potential actions can include customer-focused service delivery, collaboration across different scales of English local authority, the search for local coherence when combining EU, UK, and local government instruments, and local government contracts with the UK Treasury to connect local delivery to financial rewards for meeting national targets (2003: 162). Further, broad objectives can include ‘service improvement’, ‘community leadership’ keeping ‘in touch’ with citizens, ‘local accountability’, and ‘public confidence’ measures which lead to incoherent local packages of reform (2003: 165).

Third, *anticipate obstacles when existing commitments undermine new policy priorities.* For example, the WG pursuit of data sharing across the public sector conflicted with EU-wide data protection rules (6 *et al.*, 2005; Bellamy *et al.*, 2005; Combe, 2009; Higgs *et al.*, 2003; Higgs *et al.*, 2005; Signoretta & Craglia, 2002: 71–2; contrast with McGuirk *et al.*’s 2015).

Fourth, *the absence of clear new directions will prompt organisations to rely on old rules.* Dunlop and Russel (2012) find that vague government ambitions and messy signals to public bodies prompted regulators to fall back on their own rules. The unintended consequence was the lack of integration of the goals or performance measures among multiple regulatory agencies, contributing to “frequent ‘turf wars’” ( Cope & Goodship, 1999: 3; Goodship & Cope, 2001: 44).

Fifth, *expect ill-resourced top-down initiatives to have limited local impacts.* Numerous examples of modest impacts include on local community development, economic regeneration, strategic partnerships, health and social care integration, and performance management ( Catney, 2009: 62–3; Darlow *et al.*, 2007: 127; Exworthy & Powell, 2004: 276; Holman, 2013: 82; Lindsey, 2014: 327; Martin & Downe, 2014: 43–4; Maddock, 2000: 67; McGhee, 2003: 364; Pemberton & Winstanley, 2010: 35).

Sixth, *these negative experiences contribute to lower expectations for – or more resistance to - reform.* The outcomes contribute to a general cycle of ‘fits of enthusiasm yielding to bursts of disillusion’ ( Challis *et al.*, 1988 in Easen *et al.*, 2000: 355). Joined-up-service agendas can also prompt professions to protect their values and roles rather than shared aims ( Burnett & Appleton, 2004: 34). A hierarchical conception of local partnership fuelled by short-term targetry undermines partnership development ( Maddock, 2000: 65). The adversarial and imbalanced relationship between central and local government exacerbates blame games even when the centre claims to be seeking respectful reforms ( Masson, 2006: 241–2).

*Australian and Nordic experiences of top-down approaches to PI*

Wider international examples reinforce this criticism of inflexible and ill-resourced top-down approaches (e.g. Andoh & Lee, 2018: 18; Kelly *et al.*, 2020; Schiappacasse *et al.*, 2020: 4–5). For example, the Australian federal government’s ‘vision for social inclusion’ was not well resourced ( Carey *et al.*, 2015a: 180–3; Carey *et al.*, 2015b: 167). It struggled to influence delivery and exacerbated third sector distrust of government (2015b: 176). Similarly, Hendrix *et al.* (2020: 36–7) identify the consequences of top-down Australian policies for ‘indigenous governance’: patchy short-term contracting for services undermined collaboration and coherence (see also Sidhu & Taylor, 2009: 668–9). O'Flynn *et al.* (2011: 248) relate poor progress to the ‘lack of a JUG-Supportive Architecture’, in which the government:

• Did not ‘reset authority relationships’ to allow one agency to direct another (or oblige them to contribute to common efforts) (2011: 248).
• Maintained old ways of funding, managing performance, and holding organisations to account, which reinforced silos and minimised incentives to take resources from core agency business (2011: 249).
• Centralised policy authority, undermining the ability of local actors to collaborate meaningfully with communities (2011: 249–50; see also Humpage, 2005: 47).

Nordic examples contrast national top-down initiatives with expectations for local government autonomy. Synnevåg *et al.* (2018, 2019) describe a HiAP dilemma in Norway, where the aim to institutionalise public health aims into national strategy, and see it carried out: (1) competes with the aim to foster meaningful local collaboration, and (2) threatens local professional identities if they perceive national moves as ‘health imperialism’ (see also Kågström & Dovlén, 2019: 509; Tennøy & Øksenholt, 2018: 93; 106). Holt *et al.*’s (2018: 49) study of internal Danish municipality reforms finds that top-down reorganisations to boost intersectoral action had unintended consequences, to shift people from the roles they had performed well, reduce a sense that joint meetings were fruitful, and ‘reproduce the organizational problems they are intended to overcome’.

**Sectoral dominance: some aims are more important than others**

PI may be portrayed as a win-win strategy to serve multiple policy aims, but multiple accounts highlight contestation across organisations and sectors to assign priorities. The rhetorical aim may be to find synergies across policy sectors, but some aims, such as economic growth and national security, tend to overshadow others, such as public health and climate policies.

*Economic dominance in sustainable development and public health*

Governments signal ‘sustainable economic growth’ in theory but favour economic growth in practice ( Smith *et al.*, 2014: 2627). The mainstreaming of this idea fosters an economic imperative across sectors, including environmental, and land and water use policies which are treated as potential impediments to growth rather than independently laudable aims (2014: 2628). Or, ‘sustainable development’ is led by low power units ( Coffey, 2013: 66–70). Efforts to mainstream climate action or biodiversity may be driven by the rules of a more dominant sector, such as to require a political case - for combining economic development and climate change aims - to policymakers worried about the political costs ( Kalafatis, 2018), or ‘business case’ for conservation ( Karlsson-Vinkhuyzen *et al.*, 2018: 134). This economic lens informs trade-offs in energy and climate policies. There is support for CPI in ‘oil-producing countries’ if it does not threaten the economic benefits of fossil fuel production ( Al-Sarihi & Mason, 2020: 1226; Warren *et al.*, 2016: 8–9) or in the EU if renewable energy policy does not undermine ‘energy security’ or ‘rural economic development’ ( Rietig, 2019: 228). Similarly, water advocates are subordinate to more powerful energy actors in Jordan ( Al-Masri *et al.*, 2019: 200) and the EU ( Abazaj *et al.*, 2016) or economic aims in China ( Sun *et al.*, 2019).

In HiAP studies, the priority of economic growth, combined with a preference for markets and personal responsibility, overshadows the case for state responsibility for population health ( Cairney *et al.*, 2021a). Accounts describe the disproportionate power of industries to block WG strategies based on public health evidence ( Baggott, 2010; Battams & Townsend, 2019; Butler, 2009; Carey & Harris, 2016; Labonté *et al.*, 2018; Lencucha *et al.*, 2015; Lencucha & Thow, 2020; Moschitz, 2018; van Eyk *et al.*, 2019). The strongest contrast between vague support for HiAP as a *collaborative process* versus a *vehicle for major policy change* is in health equity ( Cairney *et al.*, 2021a; van Eyk *et al.*, 2017). Some research highlights WG obstacles whenever some actors are involved (e.g. tobacco interests), policy change is incremental (2015: 847), or public health actors are key to health departments but peripheral to economic and industry departments ( Butler, 2009: 344; Clarke *et al.*, 2021; Lencucha & Thow, 2020).

These experiences lead many public health actors to object to governments collaborating with industry, which can prompt further conflict across actors and departments (e.g. food regulation – Shill *et al.*, 2012: 168; Trevena *et al.*, 2015). Or, public health research identifies too few influential actors promoting health aims, such as when ‘nutrition’ is marginalised in foreign food trade ( Baker *et al.*, 2019: 2329; Friel *et al.*, 2019; Reeve *et al.*, 2018; Ruckert *et al.*, 2017). In exceptional cases of success, health actors build up networking and advocacy capacity, and understand trade policymaking to maximise their impact ( Thaiprayoon & Smith, 2015; see also Thow & Priyadarshi, 2013; Thow *et al.*, 2018). However, success relates more to strategy than resource allocation ( Haber & Day, 2014: 305–6).

*Economic and agricultural dominance in the EU: sustainable agriculture and neoliberal approaches to policy coherence for development*

Multiple examples demonstrate economic and agricultural interests overshadowing EPI or CPI (or sustainable transport - Dyrhauge, 2014: 985). Alons (2017: 1604–08) describes a rise in ‘environmental discourse or rhetoric’ and ‘significant changes’ to the EU Common Agricultural Policy (CAP), but a weak model of integration ‘due to low priority of environmental issues and a closed agricultural policy network’ (see also Feindt, 2010). Most substantive reforms were a by-product of economic considerations ( Alons, 2017: 1612) or small-scale local initiatives ( Buizer *et al.*, 2016; see also Huttunen, 2015). Few actions had strong positive effects on biodiversity ( Šumrada *et al.*, 2020). Further, the impact of EU water directives on agriculture is mediated through member states: the EU favours hard measures, but states use voluntary measures and guidance to influence agricultural actors ( Wiering *et al.*, 2020: 15). More generally, climate and agricultural strategies have ‘co-existed’ rather than been integrated ( Schmidt, 2020: 908; see also Schmidt & Fleig, 2018).

EU studies identify similar drivers against PCD in relation to business interests and migration policies ( Adelle & Jordan, 2014: 388–9). PCD has few powerful advocates and often struggles to gain political traction in practice ( Carbone, 2008; Carbone & Keijzer, 2016: 39–40; de Roeck *et al.*, 2018; Fritz & Raza, 2017; Guerrero & Castañeda, 2021; Larsen & Powell, 2013; Pilke, 2016; Prontera, 2016: 313; Stroß, 2017; Verschaeve & Orbie, 2018; Vieira & Lange, 2012; Zeigermann & Böcher, 2020; compare with Koch, 2018; European Commission, 2019). EU policies on migration management and PCD foster ‘neoliberal’ free trade ideas, as well as migration control, international security, and stability, over economic and social development in African states ( Koff, 2020; Schmitz & Eimer, 2020; Sørensen, 2016; Stocchetti, 2016; Thede, 2013). In such contexts, Horký, (2010: 249) describes two strategies: to criticise more powerful actors for blocking development policy, or emphasise “’win-win’ situations and new synergies”. However, the EU fosters ‘coheritization’: welcoming unthreatening criticism and sidelining harsh critics (2020: 643; compare with Radaelli, 2023).

*The dominance of foreign policy and national security*

At a wider international level, dominant aims relate largely to foreign/defence policy (see also Cairney, 2025b on examples including tourism and WGA). The starting point is *high attention* and unresolved conflict, such as regarding the purpose of international peacebuilding integration efforts (e.g. Calabrese, 2020: 307; de Coning & Friis, 2011: 263; Hu, 2020). Here, problems of coherence could reflect shifts in the balance of power internationally, or new responses within countries following policy learning or elections (2011: 270–2; Dafinova, 2021: 460–63; Ekhaugen, 2022; Leprince, 2013: 368–9; Marten, 2010). Further, PI barriers relate to *low attention*, such as when governments use the WG language insincerely to signal foreign policy actions without expecting follow through, or to downplay the fact that they prioritise domestic national security rather than peace and prosperity in foreign states ( Baranyi, 2014: 171; Desrosiers & Lagassé, 2009: 659–60; Ekhaugen, 2019; Ekhaugen, 2022: 685–6; Gabler, 2010; Johanson, 2017: 103; Olsen, 2013: 1839; Saideman, 2017: 135).

## Discussion

The search for practical lessons from policy integration and policy coherence research feels like a fool’s errand, since the literature consists largely of (1) interdisciplinary research on how policymaking should work, with few clarifying their approach to PI or drawing on political science to inform feasible aspirations or advice, then (2) political science research on how policymaking works, with few focusing on how it should work or offering explicit lessons for practice. The result plays out in a series of tables in which: there are many terms to describe aspects of PI ( Table 2), the purpose of PI and the preferred means to foster policy coherence remains unclear and contested ( Tables 3– 5), and PI research identifies some facilitators ( Table 6) but far more barriers ( Table 7). The overall lesson for academic research may be that: vaguely expressed aspirations for more integrated policy processes and coherent policies are worthy but unproductive unless they take seriously the evergreen barriers to PI, but political science research needs to step up to offer more practical advice. The overall lesson for practice is to beware the vague promotion of policy integration and coherence, regardless of its source: the good policy actors who have not interrogated their own aspirations, bad actors who are hoping that you don’t interrogate their claims, or experienced, pragmatic, or stoical actors who know that vague high level aspirations may be the best they can do if they need many country and sectoral interests to agree. In that context, while I can present a more detailed overview of lessons below, these lessons are less specific and less practical than in the two previous reviews ( Cairney & Toomey, 2024, 2025).

### General lessons for policy integration and policy coherence

Table 8 summarises a long list of lessons, which I use to produce an integrated account of the field and coherent story about how to proceed.

**Table 8.** Cautionary tales to inform policy integration.

Theme	Problem to address	Practical advice and actionable lessons
PI and WG lack clarity	PI is a broad umbrella term sheltering many empirical and normative approaches	Describe what term to use, for what purpose Process aims include to coordinate policymaking. Policy aims include to produce a coherent policy mix
Too many studies and strategies describe aspirations, not feasible plans	Too much research exhorts WG without clarity Too many governments signal tokenistic support Success stories credit WG without a clear link between approach and outcome	Define WG to include the: Rationale and priority Level and scale of inclusion Means and intensity of coordination Policy problem, timeframe, and measure of success Produce a clear theory of change
There is no best way to pursue coherence	There is no single clear, well-understood, and manageable definition of policy coherence The list of requirements resembles an ideal-type Few studies identify a coherent and deliverable policy mix	Ask: coherence from what perspective? Top-down favours strategic coherence and faithful implementation Bottom-up favours flexibility and collaboration Specify a policy mix connecting goals to instruments and intended outcomes
There is no single, self-evidently good PI model	Integration and coherence are not self-evidently good There are competing modes, and trade-offs between strategies Success measures are unclear	Show how integration and coherence would benefit policymaking and policy Clarify the value of hierarchy and networks Connect high and low level, formal and informal, strategies Establish which proxies for success matter
There is some guidance on PI	Facilitators include vision, leadership, boundary spanning, and a supportive architecture	Produce a clear vision for policy change Boost collaborative Capacity Empower actors to cross boundaries Combine hard and soft measures
Most guidance emerges from cautionary tales	Policymaking fragmentation and policy incoherence are features, not bugs If ill-prepared, the barriers to PI could become overwhelming Assigning priorities and measuring success are political choices to be confronted	Identify realistic expectations Clarify aims and means Anticipate how new rules and instruments will interact with established policy Support PI with political, financial, and organisational resources Learn from experiences of the unintended consequences of top-down approaches Establish clear priorities Connect political, process, and programmatic measures of success

### If making a commitment to policy integration, explain what it means

Policy research and practice share a common focus on vague aspirational concepts to foster policy integration in the service of policy coherence. When scholars use PI as an umbrella term, it shelters many empirical and normative approaches. Most articles describe high ambition but not a clear and substantive approach to strategy, instruments, delivery, or outcomes.

Most governments use WG rhetorically and ambiguously, without explaining what it would mean in practice, how it would change practices, the capacity to deliver, what success would look like, or how a typical citizen could learn about progress. This problem runs so deep that a rhetorical commitment could signal sincere and substantive commitment or the opposite.

### To explain what PI means, identify your rationale, preferred model, and theory of change

This rationale can combine multiple aims in a coherent origin story, such as a desire to reassert central government control to reduce fragmentation and address wicked problems. It also highlights dilemmas when seeking top-down control *and* local collaboration inside and outside of government. Therefore, establish clear priorities rather than muddle through with contradictory rules and expectations. This push for clarity informs design, to establish the complexity of the problem, the actors to include, at what levels of government, the intensity of coordination, and how to define and measure success. Produce a theory of change, from statement of commitment to intended outcomes.

### Provide a coherent account of the pursuit of policy coherence

It is possible to produce a superficially coherent story, where the aim is to: maintain consistent policymaking rules and procedures, keep a well-defined and high-priority problem high on the agenda, combine values and evidence, involve all relevant actors, coordinate across levels of government, and ensure that a coherent strategy translates into an effective policy mix in design and delivery. However, there is a clear gap between this aim and reality, and unresolved dilemmas regarding choices:

1. To favour a top-down approach to coherence, emphasising the faithful implementation of a coherent high-level strategy, or a bottom-up approach, emphasising the autonomy to collaborate and flexibility to tailor policy to local contexts. These co-existing models can complement or contradict each other.
2. To establish which sectors or objectives are the priority. The pretence is that every sector or interest can be involved. The reality is that some matter more than others. Without an explicit discussion of trade-offs and priorities, many actors will be involved then disappointed then demoralised.

### Explain why this approach has advantages over a reasonable alternative

Do not take integration to be self-evidently valuable. Acknowledge key trade-offs. The first is between specialisation and collaboration, requiring a clearer sense of how they combine in theory, and obstacles to collaboration in practice. The second is between modes of PI, such as hierarchy, collaboration, and market mechanisms. The hope is to design a mutually reinforcing mix, to provide direction and resources, support collaboration, and encourage innovation. The fear is that ill-resourced top-down approaches will provide insufficient direction and undermine bottom-up innovation and collaboration, or that specialisation and narrow collaboration in subsystems will thwart wider aims.

### Identify PI facilitators from studies of success

The literature highlights general facilitators: collaborate to produce a vision; use leadership to provide direction, boost the authorising environment, and support collaborative skills; nurture boundary spanners; and, maintain a ‘supportive architecture’ combining ‘hard’ and ‘soft’ measures.

### Use studies of failure or limited progress to understand and respond to PI barriers

Most research provides insights to learn from dispiriting experience and inform repeated efforts. It helps to identify realistic expectations, especially when finding vague aspirations backed by limited resources, or a fragile superficial consensus. There are examples of entrepreneurs finding windows of opportunity for change, but to initiate rather than deliver long-term reforms. Reformers should avoid seeing PI as design from a blank page. They face a legacy of competing rules and norms and fragmentation and competition among venues responsible for policymaking and delivery. They layer new rules and instruments onto old. In strategy, they describe high ideals combining multiple sectoral aims. In practice, they prioritise economic and national security aims.

### Attend to clarity, congruity, and capacity

In a nutshell, the aim is to boost the 3Cs:

• Clarity. Establish the aims and means of PI, attending to trade-offs, setting priorities, and establishing mechanisms for monitoring progress and fostering accountability.
• Congruity. Identify how to make new PI efforts consistent with government business, or how reforms will shift rules, expectations, and priorities. Ensure that PI efforts are popular, credible and legitimate, and likely to produce improved social and economic outcomes.
• Capacity. Assign resources to reform, including political support and strategic direction, and budgets and organisational support for innovation and collaboration.

### Take these cautionary tales seriously. Don’t wish them away then reinvent the wheel

This pursuit of clarity, congruity, and capacity is based on cautionary tales and aspirational advice: we know that the 3Cs matter because their absence has undermined reforms ( Cairney *et al.*, 2022b). WG efforts are notoriously vague and tokenistic. Governments have struggled to reconcile new and old aims and rules, and to combine the benefits of top-down direction and bottom-up collaboration. They have underappreciated the resources required to translate coherent strategies into positive outcomes. There are too many studies, identifying a stark gap between the coherence of broad strategies versus real-world delivery, to think that a single top-down strategic statement will produce coherent results. Treat integration processes as a continuous cycle of activity to identify the scope for better coordination and policy integration, *and* the lack of synergy and push to integration, then reflect on how to make repetitive efforts produce cumulative results.

## Conclusion

The PI literature ranges from broad aspiration to substantive progress:

1. Wouldn't it be nice if the government had a WG approach to this problem? I will leave it to others to define this approach.
2. Wouldn't it be nice if the government had a WG approach? Here is what it might look like.
3. The government has signalled a WG approach, but vaguely, with no substance.
4. The government has signalled a WG approach but did not overcome barriers.
5. The government has signalled a WG approach, did it well, it went well, and here are positive lessons.

Most studies are in categories 1–3: researchers and governments express the need to promote policy integration, and high hopes for success, but without demonstrating what integration or success would look like. The result is advice that seems sensible because it is vague. It informs a speculative theory of change: if you engage in this activity, you should see these results. More sophisticated advice is based on a far smaller number of context-specific studies of progress (category 5). The remaining studies are category 4, to highlight barriers and the limited willingness and ability of governments to overcome them. If we seek actionable advice from this vast literature, it must be built as much on cautionary tales as success stories, summed up as:

• If making a commitment to policy integration or a whole-of-government approach, explain what it means
• To explain what PI means, identify your rationale, preferred model, and theory of change
• Provide a coherent account of the pursuit of policy coherence
• Explain why this approach has advantages over a reasonable alternative
• Identify facilitators from studies of success
• Use studies of failure or limited progress to understand and respond to barriers.

Expressing advice in this way has three main advantages. First, it anchors new aspirations to experience. If this advice seems obvious, but still needs to be shared, it provides a wake-up call to reformers who think that expressing a rhetorical commitment to WG has any substantive weight. There is too much faith in process-based aspiration. Too few studies describe the substance of the policy instruments to be coordinated, the overall policy mix to deliver, the trade-offs to address, and the means to address inevitable integration and coherence gaps.

Second, it connects empirical experience to insights from policy theories. A too-high proportion of the literature presents an image of policymaking that resembles an ideal-type of centralised policymaking, as if coherence could be related to a single brain and coordination to one authority. Policy theories remind us that real world processes are not so amenable to coherence and control: incoherence reflects uncertainty and ambiguity, and fragmentation is a feature of policymaking systems to which to adapt.

Third, it places political dilemmas and choices at the heart of PI studies, such as when seeking a balance between top-down direction and bottom-up autonomy. When debating incoherence or unintended consequences, some blame excessive centralisation while others blame a lack of strategic clarity and direction from the top. The point is to recognise the trade-offs between top-down and bottom-up approaches, then ensure that Goldilocks-style balance is consistent rather than a fudge between aims.

These results are not entirely dispiriting, but they warn against a naïve expectation that attempts to foster better processes produce better policies. They remind us that the search for integration and coherence is a political process and exercise of power to produce winners and losers, in a complex policymaking system over which no government has full understanding or control. This point may be self-evident to political scientists. However, it is essential to a field characterised by a vague aspirational language that can make political problems seems like technical problems to be solved or temporary conflicts to be resolved through collaboration. This review shows that such wishful thinking will get us nowhere.

## Ethics and consent

Ethical approval and consent were not required.

## Dedication

The late Professor Gemma Carey was a key contributor to this field, and her work has its own section in this paper. See Dickinson *et al.* (2025) on Gemma’s contribution to policy scholarship, advocacy, practice, and our profession.

## Data Availability

No additional data are associated with this article. Open Science Framework: Policymaking integration ( Cairney, 2025a) https://osf.io/hrbm2/ This project contains the following extended data:
− Initial report (36000 words) and full and structured bibliography ( Cairney, 2025b) https://osf.io/zjngf
− Structured bibliography https://osf.io/abt29
− Study Protocol https://osf.io/m8rbw Data are available under the terms of the Creative Commons Attribution 4.0 International license (CC-BY 4.0) ( Cairney, 2025a). Open Science Framework: Policymaking integration https://osf.io/hrbm2/ (DOI 10.17605/OSF.IO/HRBM2) The project contains the following additional information: PRISMA checklist for Policymaking integration, policy coherence, and whole-of-government approaches: a qualitative systematic review of advice for policymakers https://osf.io/m2uzs Data are available under the terms of the Creative Commons Attribution 4.0 International license (CC-BY 4.0) ( Cairney, 2025a).
