# Supplementary material for: Policy integration, policy coherence, and whole-of-government approaches: a qualitative systematic review of advice for policymakers
Source: Open Res Eur. 2026 Jul 9;5:75. Originally published 2025 Mar 21. [Version 2] doi: 10.12688/openreseurope.19864.2 (PMC12489461; doi:10.12688/openreseurope.19864.2)
Supplement: Peer review history

# Reviewer report: Reviewer response for version 1

**DOI:** 10.21956/openreseurope.21490.r52376

**Published:** 2025-05-24

**Authors:** Andrew J Jordan

**Affiliations:** 1 School of Environmental Sciences, University of East Anglia, Norwich, England, UK

I really enjoyed reading this paper.  It addresses an important gap in the academic literature AND it speaks directly to the ongoing debate amongst policy makers about the need for (and also the immense difficulty of accomplishing) greater policy integration.  It confidently navigates the swamp of competing terms (policy integration, mainstreaming, joined up government etc.) which easily trip up the unwary and the downright naïve. Although I have contributed extensively to some of the sub literatures that this article systematically reviews, I learnt many new things (and had some existing suspicions powerfully confirmed).  I look forward to using variants of Fig 3 and Table 8 in my teaching (both for students and policy makers).

 I have two suggestions on how to improve it (and apologies – the web version I am working from does not provide page numbers):

 1. The narrative flow is strong until the reader arrives at the case studies/country examples.  Thereafter, it weakens (i.e. starting with Barriers to JUG in the UK and running through General Barriers then arriving at Domestic Examples).  The material is really helpful (policy makers, in my experience, are always eager to know what other countries are up to), but it could be structured/ordered/signposted in a much more logical fashion.

 2. The section entitled: “Discussion: Lessons for policymaking integration and policy coherence”: first of all, I would delete the word ‘Discussion’.  Second, it would be very useful to spell out the lessons for academics.  After all, this is a literature that has often remained ensnared in the Policy Making Cycle (Figure 3), uncritically accepting repeated policy maker demands for ‘more coherence’ in ever more policy fields.

Are sufficient details of the methods and analysis provided to allow replication by others?

Yes

Are the rationale for, and objectives of, the Systematic Review clearly stated?

Yes

Is the statistical analysis and its interpretation appropriate?

Yes

Are the conclusions drawn adequately supported by the results presented in the review?

Yes

Reviewer Expertise:

Public policy, political science

I confirm that I have read this submission and believe that I have an appropriate level of expertise to confirm that it is of an acceptable scientific standard, however I have significant reservations, as outlined above.

# Response

**Published:** 2026-06-16

**Authors:** Paul Cairney

**Affiliations:** University of Stirling, UK

**Reply to Andrew J Jordan** Thank you for your positive and constructive review. I have made the following revisions:

- Improve the narrative flow by the time ‘the reader arrives at the case studies/country examples/  Barriers to JUG in the UK and running through General Barriers then arriving at Domestic Examples. I have addressed the flow problem, caused largely by my insertion of a huge section (with country/sectoral examples) in a confusing place. I moved up the ‘Unclear accountability and competing measures of success’ section to stop it getting lost at the end of a large discussion. I marked more clearly the country examples and sectoral examples.
- Delete the word ‘Discussion’  and: ‘spell out the lessons for academics.  After all, this is a literature that has often remained ensnared in the Policy Making Cycle (Figure 3), uncritically accepting repeated policy maker demands for ‘more coherence’ in ever more policy fields’. I keep the ‘Discussion’ heading (for the sake of systematic review format) but keep it separate from the sub-heading, then provide some more reflection on advice for academics and practitioners (the Conclusion makes these points a bit more strongly).

# Reviewer report: Reviewer response for version 1

**DOI:** 10.21956/openreseurope.21490.r52367

**Published:** 2025-04-30

**Authors:** Philipp Trein

**Affiliations:** 1 University of Lausanne, Lausanne, Switzerland

This report is based on a review of the literature on policy integration, policy coherence, and whole-of-government approaches. The author conducts an in-depth analysis of different strands of literature that address how policymaking systems respond to complexity. The key takeaway from the review is that practitioners should focus on the three Cs—clarity, congruity, and capacity—when it comes to policy integration. Moreover, the author encourages the literature to emphasize success stories and positive examples of policy integration, rather than focusing primarily on barriers, in order to better engage decision-makers.

 Overall I like this report very much it's very successfully reports different aspects of the vast literature that comes along with a lot of conceptual diversity and very different empirical approaches we'll start there also agreed that we lack a number of success stories concerning policy integration but this might be because it's unclear whether such form of holistic policy making have above all political value by showing that governments think of problems in an encompassing way or whether they're also live that deliver better results than more directed sectoral interventions. In revising the paper suggests that the authors take into account the the following comments.

 Firstly, I reviewed the author's work, and I believe the author should clarify the term policymaking integration. I assume that the author refers not only to incorporating different sectors and levels of government into a problem-solving approach, but also to promoting greater coherence between agenda-setting, decision-making, and implementation. My concern is that this term adds yet another layer of complexity to an already vast number of existing concepts. I wonder whether it might be more effective to repurpose one of the existing terms as an overarching concept. Furthermore, the conceptual literature on policy integration has defined policy integration as a process. How is policymaking integration different from that?

 Secondly, the concept of coherence needs to be defined more clearly. The starting point in the literature for this review focuses on diagnosing coordination problems between departments, holistic approaches to policy issues, and the recentralization of government through joined-up or whole-of-government initiatives. However, the author appears to explore coherence more broadly, particularly between different stages of the policy process. This aspect should be articulated more explicitly, as it diverges from the direction suggested by the initial body of literature. The diagnosis that policymakers operates in contexts of bounded rationality and non-linear processes that are difficult to predict is something that emerges from other public policy scholarship.

 While the concept of policy integration is central to this text, it does not address the notion of vertical policy integration, which has recently gained prominence. This concept highlights the potential gap and incoherence between decisions made at the top and implementation actions taken at the local level. By engaging with this additional literature, the author could better address one of the key challenges of policy coherence: the disconnect between the formulation of broad goals and the lack of corresponding implementation efforts.

Are sufficient details of the methods and analysis provided to allow replication by others?

Yes

Are the rationale for, and objectives of, the Systematic Review clearly stated?

Yes

Is the statistical analysis and its interpretation appropriate?

Yes

Are the conclusions drawn adequately supported by the results presented in the review?

Yes

Reviewer Expertise:

NA

I confirm that I have read this submission and believe that I have an appropriate level of expertise to confirm that it is of an acceptable scientific standard, however I have significant reservations, as outlined above.

# Response

**Published:** 2026-06-16

**Authors:** Paul Cairney

**Affiliations:** University of Stirling, UK

**Reply to Philipp Trein** Thank you for your positive and constructive review. I have made the following revisions:

- Use the established term ‘policy integration’ and explain why I use ‘policymaking integration’. I now use ‘policy integration’ as the default term in the text and title and have restricted the term ‘policymaking integration’ largely to the section that describes the analytical distinction between policy process and policy analysis elements of integration (in relation to policymaking integration and policy coherence, above and below Table 2).
- Explain this review’s approach to policy coherence (subheading ‘What exactly is policy coherence?’). I revise the opening paragraph to describe the expansion of this literature and the unwieldy results. I now explore this topic more fully in a separate article published in Policy Sciences (now cited).
- Note the term ‘vertical policy integration’ and highlight the ‘the potential gap and incoherence between decisions made at the top and implementation actions taken at the local level’. I expand on the *Top-down and bottom-up perspectives *paragraph discussing policy coherence, and refer to vertical PI in that discussion. My discussion of the more general tension between competing forms of PI is mainstreamed throughout the article (and I add a sentence to advise readers to check what each author means by ‘vertical’).

# Reviewer report: Reviewer response for version 1

**DOI:** 10.21956/openreseurope.21490.r52370

**Published:** 2025-04-10

**Authors:** Muiris MacCarthaigh

**Affiliations:** 1 Queen's University Belfast, Belfast, Northern Ireland, UK

This is a very ambitious and welcome effort to bring together some tricky ideas (policy integration, and policy coherence) in a meaningful way, and to manifest what may often appear to be very general or all-encompassing concepts with practice. The paper sits alongside two previous review papers, and all are part of a wider project which specifically involves policy coherence. The author’s plain use of language and deliberate unpacking of jargon is very helpful, and I particularly liked the observation (on p.7): ‘The study and practice of integration and coherence lacks integration and coherence’. The article itself (and particularly Table 2 and the bibliography) provides an excellent primer for students of public administration and management.

 The summary and well-evidenced account of how the concepts under examination have waxed and waned is very well written. It also speaks to a core finding that the literature has a bias towards describing the antecedents necessary for something to work, rather than examinations of successful or unsuccessful examples of integration for coherence. The focus on the UK case in the paper is useful, and reflects the authors’ extensive knowledge of that system. There is some attention given to a variety of cases from across the European Union and internationally but these are brief.

 The method used is a well-established one, and the systematic review conducted here considered quite a large range of publications which deserves great credit (and the teams involved in this are properly acknowledged). As a result the paper presents a real tour de force of how integration and coherence, as well as various associated synonyms, have been applied in the literature and in practice. However such an extensive review can make it difficult to draw precise lessons and it seems it was not possible to apply a quantitative approach to distil other insights. Therefore the lessons for practitioners are a little basic and general, if well-informed.

 The overall analysis is judicious and does well to demonstrate the pros and cons of different perspectives on policy integration and to distinguish between it as a process as opposed to an end in itself.

Are sufficient details of the methods and analysis provided to allow replication by others?

Yes

Are the rationale for, and objectives of, the Systematic Review clearly stated?

Yes

Is the statistical analysis and its interpretation appropriate?

Yes

Are the conclusions drawn adequately supported by the results presented in the review?

Yes

Reviewer Expertise:

Public Administration, Public Policy, Politics

I confirm that I have read this submission and believe that I have an appropriate level of expertise to confirm that it is of an acceptable scientific standard.

# Response

**Published:** 2026-06-16

**Authors:** Paul Cairney

**Affiliations:** University of Stirling, UK

**Reply to Muiris MacCarthaigh** Thank you for your positive and constructive review. I have made the following revision:

- Discussion. I reflected on this feedback ‘the lessons for practitioners are a little basic and general’ and sought to provide better lessons or explain their absence. The latter option won.

# Reviewer report: Reviewer response for version 1

**DOI:** 10.21956/openreseurope.21490.r52374

**Published:** 2025-04-01

**Authors:** Helen Dickinson

**Affiliations:** 1 University of New South Wales, canberra, Australia

This paper examines the literature to gather data about how to foster policymaking integration in the service of policy coherence. This is a large search of the literature involving over 700 texts. This is well-trodden academic ground and a number of systematic reviews have been undertaken. This study does not seek to do another systematic review but to provide additional analysis on the studies identified and to add insights from the grey literature to this. The analysis seeks to find pragmatic advice that offers hope to policy markers but to also acknowledge constraints on policymakers. The aim of the paper is to provide actionable lessons, although not a how-to-guide. In this sense it is novel in the context of the existing literature. 

 This paper is part of a broader programme of work and the link between this study and the broader project is well described. 

 I am not sure that some of the material in the methods section is in the right place. This section describes the method and then concludes with the key themes found by Aoki et al (2024a) and the two Trein et al papers. I wondered whether this section would be better placed either in the introduction or as initial part of the findings section. The section on analysis is quite limited and a bit more information on the decisions made in analysing the data would strengthen the paper. I am interested particularly in how the guiding questions interacted with the ways that themes were inductively extracted from the various sources. 

 This is a bold paper but the findings sections do an excellent job of bringing together many of the key features that are pertinent to WG/JG and its implementation. Cairney does an effective job of creating a number of analytical tools in the form of tables that will be useful in practice to policy makers who are developing or assessing a range of integration initiatives. The account of the literature feels to be exhaustive and does not just focus on higher level issues as many studies do, but really gets into the nuance of these many different accounts that shed light on different facets of policy integration. 

 The discussion sets out a number of lessons to inform policymaking integration. These are largely sensible and are consistent with what has been set out in the findings section. I did have one reflection in looking at these that they are largely concerned with identifying what an integration should achieve and the assumptions that underpin this. While I think in some senses this is sensible, it does largely suggest that one integrated and coherent model is possible and that this might prevent some of the more performative approaches to WG/JG that are found in the literature. On the former it may take some considerable work to get to one version and on the latter we know this is an inevitable feature of contemporary public policy and public administration practice. Having said this. Cairney does acknowledge that integration is a continuous cycle rather than an event - offering possibility for eventual gains in processes that often require significant and repetitive iterations of reform for them to make a difference. 

 Typos

 In the section on a lack of capacity ‘if so, there is always be a gap between PI strategy and deliver’ think ‘is’ should be ‘will’.

Are sufficient details of the methods and analysis provided to allow replication by others?

Partly

Are the rationale for, and objectives of, the Systematic Review clearly stated?

Yes

Is the statistical analysis and its interpretation appropriate?

Yes

Are the conclusions drawn adequately supported by the results presented in the review?

Yes

Reviewer Expertise:

public policy, public administration, collaboration in public policy, disability policy and programmes

I confirm that I have read this submission and believe that I have an appropriate level of expertise to confirm that it is of an acceptable scientific standard, however I have significant reservations, as outlined above.

# Response

**Published:** 2026-06-16

**Authors:** Paul Cairney

**Affiliations:** University of Stirling, UK

**Reply to Helen Dickinson** Thank you for your positive and constructive review. I have made the following revisions (or replies about suggested revisions):

- Reconsider what data should appear in Methods. I have retained the ‘routine data collection’ section (summarising Trein/Aoki background data) in Methods to maintain consistency across the series of systematic reviews and allow for immediate reflections on limitations. I have kept this background analysis to a minimum, largely to signal the skewing of articles to a small number of countries.
- Describe the inductive and analytical approach in more depth. I added a large paragraph in *Data analysis, aggregation, and presentation* to expand on the description of my analysis (‘I am interested particularly in how the guiding questions interacted with the ways that themes were inductively extracted from the various sources.)
- Typos. I address the typos – thank you for spotting them.
